# Immuno-genomic landscape of osteosarcoma

**DOI:** 10.1038/s41467-020-14646-w

**Published:** 2020-02-21

**Authors:** Chia-Chin Wu, Hannah C. Beird, J. Andrew Livingston, Shailesh Advani, Akash Mitra, Shaolong Cao, Alexandre Reuben, Davis Ingram, Wei-Lien Wang, Zhenlin Ju, Cheuk Hong Leung, Heather Lin, Youyun Zheng, Jason Roszik, Wenyi Wang, Shreyaskumar Patel, Robert S. Benjamin, Neeta Somaiah, Anthony P. Conley, Gordon B. Mills, Patrick Hwu, Richard Gorlick, Alexander Lazar, Najat C. Daw, Valerae Lewis, P. Andrew Futreal

**Affiliations:** 1 Department of Genomic Medicine, The University of Texas MD Anderson Cancer Center, Houston, TX USA; 2 Department of Sarcoma Medical Oncology, The University of Texas MD Anderson Cancer Center, Houston, TX USA; 3 Department of Pediatrics, The University of Texas MD Anderson Cancer Center, Houston, TX USA; 4 Department of Bioinformatics and Computational Biology, The University of Texas MD Anderson Cancer Center, Houston, TX USA; 5 Department of Thoracic/Head & Neck Medical Oncology, The University of Texas MD Anderson Cancer Center, Houston, TX USA; 6 Department of Pathology, The University of Texas MD Anderson Cancer Center, Houston, TX USA; 7 Department of Biostatistics, The University of Texas MD Anderson Cancer Center, Houston, TX USA; 8 Department of Systems Biology, The University of Texas MD Anderson Cancer Center, Houston, TX USA; 9 Department of Orthopedic Oncology, The University of Texas MD Anderson Cancer Center, Houston, TX USA

**Keywords:** Cancer genomics, Immunology, Bone cancer

## Abstract

Limited clinical activity has been seen in osteosarcoma (OS) patients treated with immune checkpoint inhibitors (ICI). To gain insights into the immunogenic potential of these tumors, we conducted whole genome, RNA, and T-cell receptor sequencing, immunohistochemistry and reverse phase protein array profiling (RPPA) on OS specimens from 48 pediatric and adult patients with primary, relapsed, and metastatic OS. Median immune infiltrate level was lower than in other tumor types where ICI are effective, with concomitant low T-cell receptor clonalities. Neoantigen expression in OS was lacking and significantly associated with high levels of nonsense-mediated decay (NMD). Samples with low immune infiltrate had higher number of deleted genes while those with high immune infiltrate expressed higher levels of adaptive resistance pathways. *PARP2* expression levels were significantly negatively associated with the immune infiltrate. Together, these data reveal multiple immunosuppressive features of OS and suggest immunotherapeutic opportunities in OS patients.

## Abstract (web summary)

The efficacy of immune checkpoint inhibitors (ICI) in osteosarcoma has been limited. Here, the authors investigate the immunogenomic landscape of osteosarcoma, and integrated analyses highlight features related to a suppressed immune microenvironment.

## Introduction

Osteosarcoma (OS) is the most common primary solid tumor malignancy of the bone predominantly occurring in adolescents with a second peak in incidence amongst older adults^1^. For patients presenting with localized disease at diagnosis, standard multi-agent chemotherapy combined with surgical resection yields long-term survival rates of ~70%^1,2^. Metastatic disease either at diagnosis or at the time of recurrence portends a poor prognosis with survival of 20–30%^3,4^.

Recent whole genome sequencing (WGS) and molecular profiling studies undertaken in predominantly pediatric populations have shown high levels of chromosome structural variations, rearrangements resulting from chromothripsis (20–89%) as well as mutation clusters known as kataegis (50–85% of cases) that result in significant disease heterogeneity but few recurrent clinically actionable alterations^5–7^. These studies have yielded insights into aberrant signaling pathways such as PI3K/mTOR (24%)^7^, IGF signaling (7%)^6^, and Wnt signaling^8^. However, the efficacy of targeted therapies such as mTOR inhibitors in unselected patient populations with relapsed osteosarcoma has been limited^9,10^. The degree of genomic instability suggests that the burden of antigens and neoantigens would provide an immunogenic potential in OS. However, this rationale has not met with the expected responses in current trials using immune checkpoint inhibitors in OS^11,12^. Currently, the population of interest for developing immunotherapy in OS are patients with poor risk disease (i.e., those with recurrence or metastasis following standard chemotherapy). Here, we use multi-platform profiling in parallel on such a population, including both children and adults, to define the immunogenic potential and to propose strategies to enhance the efficacy of immunotherapy.

## Results

Our cohort consisted of patients with high-grade osteosarcoma with poor-risk (73% having unfavorable pathologic response to standard therapy) and adverse survival outcomes (67% deceased) (Table 1, Supplemental Data 1). This cohort was enriched for relapsed (13/54; 23%) and metastatic specimens (27/54; 51%). We first assessed genomic complexity within the cohort by using high-depth whole genome sequencing (WGS) on 35 specimens with matched normal (average coverage for tumor and normal were 78X and 39×, respectively) (Supplemental Data 2). These data were then integrated with transcriptome, T-cell receptor (TCR) sequencing, reverse phase protein array (RPPA), and immunostaining results to characterize the immunogenomic landscape of OS.

**Table 1:** Clinical characteristics of 48 patients with osteosarcoma.

Characteristic	*n* (%)
Median age at diagnosis in years (range)	27 (5-81)
Gender	
Male	29 (60)
Female	19 (40)
Race	
White	30 (63)
Hispanic	10 (21)
Black	5 (10)
Asian	3 (6)
Disease stage at presentation	
Localized	37 (77)
Metastatic	11 (23)
Histologic subtype	
Osteoblastic	14 (29)
Fibroblastic	9 (19)
Chondroblastic	6 (13)
Extraskeletal	6 (13)
Dedifferentiated parosteal	5 (10)
Telangiectatic	2 (4)
Giant cell rich	1 (2)
High-grade surface	1 (2)
Other—high grade	4 (8)
Pathologic response to neoadjuvant chemotherapy (*n* = 41 patients)*	
Good (tumor necrosis ≥ 90%)	11 (27)
Poor (tumor necrosis < 90%)	30 (73)
Vital status	
Alive	16 (33)
Dead	32 (67)
Specimen type (*n* = 54)**	
Primary tumor	14 (26)
Local recurrence	13 (23)
Metastasis	27 (51)
Testing platforms (number of specimens)**	
Whole genome sequencing (WGS)	35
RNA sequencing (RNAseq)	51
Reverse phase protein prray (RPPA)	38
T-cell receptor sequencing (TCR)	41
Immunohistochemistry	33

* For patients who received neoadjuvant chemotherapy

**Two samples from same tumor site in 3 patients

### Genomic landscape

**Fig. 1:**
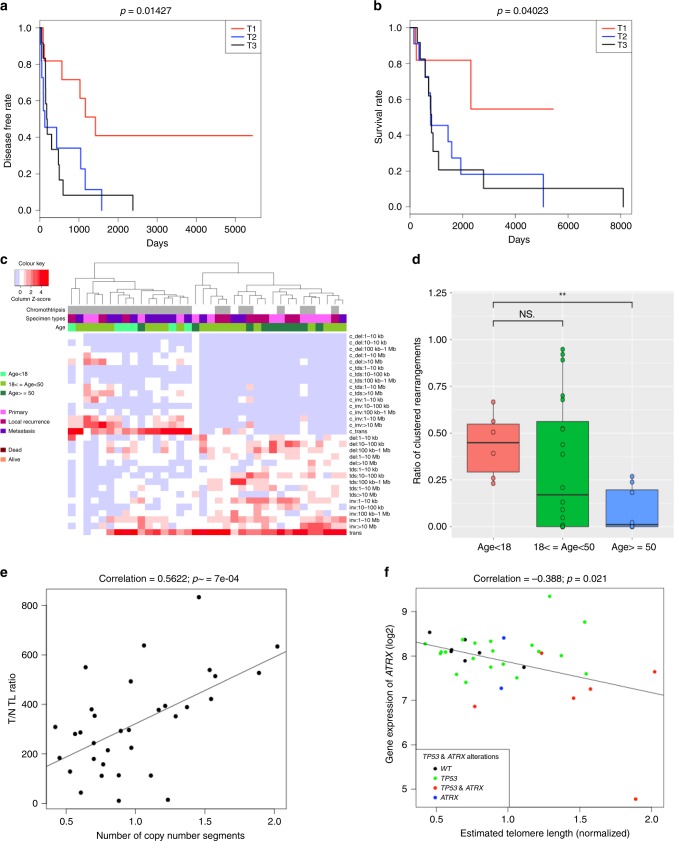
Clinical associations with somatic alterations in OS. **a**, **b** Kaplan-Meier survival analysis (disease-free survival for **a**; overall survival for **b**) of mutation signature 8 scores which describe the contribution of mutation signature 8 to the point mutation profile. The cohort was separated into tertiles based on signature 8 scores. Group 1 (denoted as T1) is the first tertile with lowest mutation signature 8 scores. Groups 2 and 3 (denoted as T2 and T3 respctively) are the next 2 tertiles with higher mutation signature 8 scores. **c** Hierarchical clustering heatmap of rearrangements classification pattern identified in our cohort along with clinical features: age of diagnosis (<18 years, 18– < 50 years, and >50 years), tumor specimen type (primary, local recurrence, metastasis), and vital status (dead, alive). Structural rearrangements were classified based on their type: deletion (del), tandem duplication (tds), inversion (inv), and interchromosomal translocation (trans), and size, and clustered (denoted as c_) versus non-clustered events. **d** Boxplot showing the ratio of clustered rearrangements within associated with chromothriptic regions as compared across three groups of specimens based on age of diagnosis (<18 years, 18–<50 years, and >50 years). Then significance values (*P*-values) of the comparisons are from the Wilcoxon rank sum test are shown in asterisks. **e** Pearson correlation between normalized telomere length and number of copy number segments of samples. **f** Pearson correlation between normalized telomere length and *ATRX* gene expression level (in log2 scale) of samples. Samples with both *ATRX* and *TP53* alterations, *ATRX* alterations alone, and *TP53* alterations alone were respectively marked as red, blue, and green color.

The genomic landscape in these largely advanced tumors was similar to what has been reported (Supplemental Fig. 1, Supplemental Data 3a-b)^5–7^. No truncating germline mutations associated with DNA repair were observed. In addition, no significant differences were seen across the primary, local recurrence, and lung metastasis specimens in mutation burden (based on nonsilent single nucleotide variants, small insertions and deletions), predicted neoantigens, subclonal proportions, copy number alterations, and altered pathways (Supplemental Fig. 2, Supplemental Data 4a-b). Nonetheless, we identified several interesting genomic features in our cohort. First, the contribution of mutation signature 8 (Supplemental Fig. 3a) was positively associated with mutation burden, disease relapse status, and survival, but not with kataegis (Fig. 1a, b). Second, the high levels of genomic rearrangements (Supplemental Fig. 1e) could be divided into two major groups corresponding to rearrangement signatures 2 (non-clustered) and 4 (clustered) (Fig. 1c)^13^. There was a trend for younger patients to have rearrangements that are clustered and associated with chromothripsis as compared with older patients, which was also observed in the Therapeutically Applicable Research to Generate Effective Treatments (TARGET) cohort that is composed of mostly pediatric OS (Fig. 1d, *P* < 0.01, Wilcoxon rank sum test; Supplemental Fig. 3b). On average, 18.9% of breakpoints occurred in fragile sites, which is comparable to the mean found in the BOCA-UK cohort (16.5%) (Supplemental Data 5). Third, OS cells may maintain viability of their *TP53/RB1* mutation-induced unstable genomes through whole-genome doubling (WGD) or telomere lengthening^6,14,15^ (Supplemental Fig. 3c). In our cohort, both WGD and normalized telomere lengths had significant positive association with high copy number and rearrangement burden (Fig. 1e *P* < 0.001, *P* < 1e-4, respectively, Pearson’s correlation). Up to 50% (18/36) of patients had losses of heterozygosity in *TP53* and/or *RB1* along with WGD (Supplemental Fig. 1a). Given the inherent lower likelihood of losing two copies after WGD, this supports that *TP53* and *RB1* aberrations likely occurred before WGD^16,17^. Genetic alterations and expression of *TERT* do not have significantly longer telomeres (Supplemental Fig. 3d). Instead, lower expression levels of *ATRX* were significantly correlated with longer normalized telomere lengths (Fig. 1f). Seven patients with deleterious alterations in *ATRX* as well as one patient with copy number loss in *DAXX* had telomere lengths greater than the cohort median (Fig. 1f). Patient samples with the longest telomere length carried alterations in both *TP53* and *ATRX*, supporting the permissive context in which *TP53* alterations can allow for activation of alternative lengthening of telomeres (ALT)^18^. In addition, the expression levels of telomere maintenance genes *HNRNPA2B1*, *WRN*, and *HUS1*^19^, were also significantly correlated with telomere length (*P* < 0.001, *P* *<* 0.05, *P* *<* 0.05, respectively, Pearson’s correlation) (Supplemental Data 6). However, the exact mechanisms surrounding telomere maintenance in the ALT pathway is unclear. Thus, effect of the telomere-related mutations on ALT are yet to be explored.

Furthermore, we also compared genomic features based upon pathologic tumor response to neoadjuvant chemotherapy as well as a comparison across histologic subtypes. We found that samples with favorable tumor necrosis (defined as ≥90% necrosis following chemotherapy) have higher COSMIC 3 signature scores, telomere lengths, and Th2 T cell scores as compared to those with poor tumor necrosis (<90%) (Supplemental Data 7). This suggests that the genomes may be less stable and chronic activation of immune response in those with favorable necrosis^20^.

### Mutation burdens were not associated with immune infiltrate levels

The high levels of genomic rearrangements and moderate point mutation burdens in OS suggests that the levels of neoantigens should be high enough to elicit an immune response^21^. Although higher nonsilent mutation burden was associated with higher number of predicted neoantigens in our cohort (Supplemental Fig. 4a), transcriptome immune infiltrate scores (ESTIMATE)^22^ were not influenced by having more neoantigens (Supplemental Fig. 4b). This was further supported by the lack of association between an RPPA immune-related markers (Caspase-7 (cleaved D198), Lck, Syk, and Pded-1L1) (see Methods) and predicted neoantigen burden (Supplemental Fig. 4c). Similarly, no relationship was found between immune infiltrate scores (ESTIMATE) and the total number of rearrangements (Supplemental Fig. 4d).

### Low level of predicted neoantigen expression

**Fig. 2:**
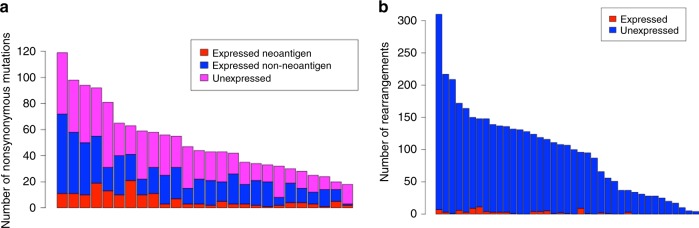
Expressed neoantigens and rearrangements. **a** Stacked bar chart for number of nonsynonymous mutations and neoantigens detected by WGS and capture-based RNASeq. (expressed neoantigen: nonsynonymous mutations found in both WGS and capture-based RNASeq that are predicted to be a neoantigen; expressed non-neoantigen: nonsynonymous mutations found in both WGS and capture-based RNASeq that are not predicted to be a neoantigen; unexpressed: found in WGS only). **b** Overlapping rearrangements between WGS and RNASeq based rearrangements where both partners are within coding regions (unexpressed: found in WGS only; expressed:found in both WGS and RNASeq).

Given the levels of rearrangements and mutation burden, we explored whether these genomic alterations are expressed and the potential levels of neoantigens. Fewer than 30% of expressed nonsynonymous changes as detected by RNAseq were predicted to be strong-binding neoantigens (5–30% per patient) (Fig. 2a). As expected, unexpressed mutations tended to occur in genes that have low expression (Supplemental Fig. 5a) or whose variant allele frequencies were <0.25, likely a limit in sensitivity due to RNASeq (Supplemental Fig. 5b). Limited overlap between point mutations identified in both DNA and RNA sequencing has been observed in non-small cell lung cancer as well as glioblastoma suggesting that this finding is not unique to OS^23,24^. In addition, few predicted rearrangements involving coding regions were expressed (Fig. 2b). Of interest, we observed a positive association between nonsense- mediated decay (NMD) factors and the number of gene-containing rearrangements as well as immune scores (*P* < 0.05, Pearson’s correlation) (Supplemental Fig. 6), suggesting that there may be substantial transcript suppression in rearranged OS genomes. Overall, despite substantial proportions of predicted neoantigenic mutations and genic rearrangements, there are few alterations ultimately expressed at high levels.

### T-cells present but low T-cell receptor productive clonality

The majority of specimens (21/33, 64%) showed medium or high density staining for CD3 + T-cells, which indicated that T-cell infiltrate was present in a most samples (Supplemental Data 8a). Lung metastases had higher CD3, CD4 T-cell and M2 macrophage (CD163) staining densities than primary specimens (*P* < 0.05, *P* < 0.01, and *P* < 0.05, respectively, Wilcoxon rank sum test) (Supplemental Data 8b-c). However, T-cell activity as estimated by productive clonality was low overall (average = 0.09) with a maximum of 0.3 in comparison to normal skin (0.15)^25^ and healthy adult female peripheral blood mononuclear cells (~0.05–0.2)^26^ (Supplemental Data 8d). Five cases (WGS_12-15, WGS_12-23, WGS_12-24, WGS_12-35, WGS_12-7) had one clone that represented the largest proportion (0.01–1) of the T-cell population (as measured by homeostatic space) and all of these had low numbers of T-cell clonotypes (<100) (Supplemental Fig. 7). These data suggest a lack of T-cell clonal diversity that may blunt the ability to mount an effective immune response.

### Insufficient immune infiltrate

**Fig. 3:**
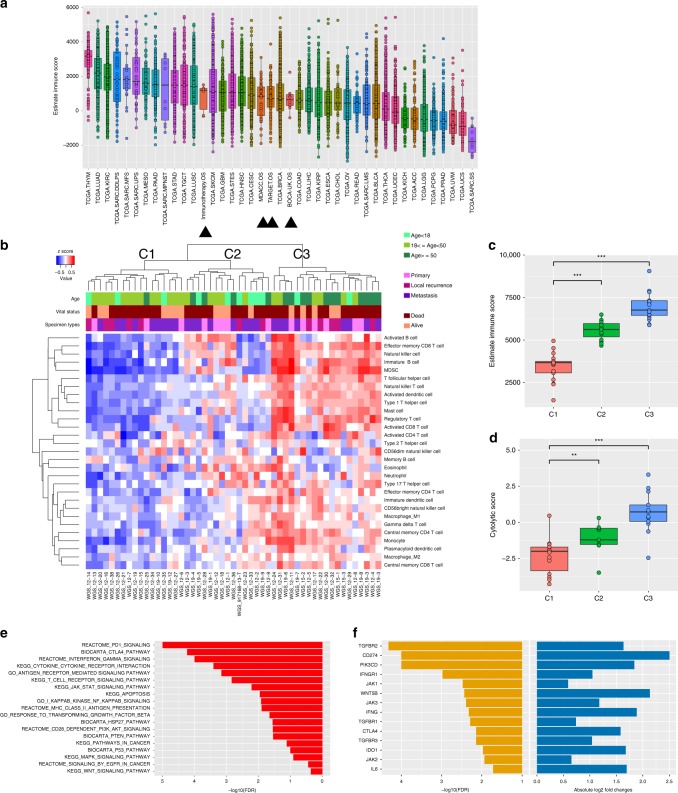
Immune profiling. **a** ESTIMATE immune scores in TCGA tumor types, our OS cohort (MDACC.OS), the OS cohort from ICGC (BOCA-UK.OS), the OS cohort from TARGET (TARGET.OS), and from four patients who underwent combination CTLA-4 blockade and PD-L1 blockade but exhibited no objective responses (Immunotherapy.OS). Note that TCGA-THYM is derived from the thymus made up mostly of lymphocytes and would have inflated scores. **b**. Unsupervised hierarchical clustering based on ssGSEA enrichment scores of each immune gene list from Charoentong et al. The three predominant clusters are referred to as “C1” and “C2”, and “C3” immune infiltrate groups. **c** Boxplot of the ESTIMATE immune scores of samples across the three immune clusters C1, C2, and C3. The significances of the comparisons were from the Wilcoxon rank sum test are shown in asterisks. **d** The geometric mean expression of *GZMA* and *PRF1* genes (cytolytic score) shown as a boxplot across the three immune clusters C1, C2, and C3. The significances of the comparisons were from the Wilcoxon rank sum test. **e** Immunosuppressive pathways that are significantly deregulated between immune clusters C1 and C3. The significance (FDR) of deregulation were from the GSEA analysis. **f** Fold change and significance of expression difference between C1 and C3 for key genes from pathways in **e**: *CD274*(PD-L1), *CTLA4*, *IDO1*, *IFNG*, *IFNGR1*, and *IL6* (these genes were not present in the gene lists used to define these immune groups). The linear mode in the limma R package was used to determine significance.

To understand the immune infiltrate level of OS in a broader context, we compared immune infiltration scores (ESTIMATE) from our cohort against other tumor types profiled in The Cancer Genome Atlas (TCGA), the OS samples from the International Cancer Genome Consortium (ICGC) and TARGET studies, as well as four additional patients with metastatic OS who were treated with combination CTLA-4 blockade and PD-L1 blockade but exhibited no objective responses (Fig. 3a). The median immune scores from the ICGC cohort, TARGET, and the four pre-treatment OS samples for patients treated with ICI were comparable to our cohort. Skin cutaneous melanoma (TCGA-SKCM), lung cancer (TCGA-LUAD and TCGA-LUSC) and renal clear cell carcinoma (TCGA-KIRC), are tumor types that have clinical benefit and response to immune checkpoint blockade related to high immune infiltrate levels whereas other tumor types are known to have very low immune infiltrate such as low-grade glioma (TCGA-LGG), prostate cancer (TCGA-PRAD), and uveal melanoma (TCGA-UVM) and have seen limited activity with current immunotherapy approaches. Within this context, we observed that specimens from our cohort have intermediate median ESTIMATE scores that are lower than melanoma and lung cancer, but higher than uveal melanoma. When compared to other sarcoma subtypes, the median immune score of dedifferentiated liposarcoma (TCGA-DDLPS) and undifferentiated pleomorphic sarcoma (TCGA-UPS)—2 soft tissue sarcoma subtypes where ICI are active—are higher than OS samples. We also examined samples whose ESTIMATE immune scores are in the highest quartile among all the samples, which may be likely benefit from the ICI. Overall, 8% (4/51) of our cohort samples, 10% (1/10) of the ICGC cohort, 15% (12/84) of the TARGET cohort, 0% (0/4) OS patients treated with ICI are in the highest quartile (Supplemental Fig. 8). These data indicate that most of OS specimens we examined may have insufficient immune infiltrate to elicit meaningful responses to ICI alone.

### Evidence for multiple immuno-modulatory mechanisms

To characterize and compare the composition of the immune infiltrate across our samples, we first generated single sample GSEA gene expression enrichment scores for various immune cell types for each patient (based on gene lists from Charoentong et al.)^27^. Hierarchical clustering on these scores revealed three clusters: C1, C2, and C3 with increasing levels of immune infiltrate: C1 having the lowest and C3 having the highest levels of all immune cell types including CD8 lymphocytes (Fig. 3b, Supplemental Fig. 9a). These significantly different levels of expression were also reflected in the immune infiltrate scores that were present (by ESTIMATE and TIMER)^28^ (Fig. 3c, Supplemental Fig. 9b-h) as well as cytolytic scores^29^ (*GZMA* and *PRF1*) (Fig. 3d). The TARGET dataset also revealed several clusters, two of which are comparable to our C1 “cold” and C3 “hot” immune infiltrate groups (Supplemental Fig. 10). For our cohort, 9 of the 14 older patients (age > 50) were in the C3 group with higher immune infiltrate levels (Fig. 3b). This finding corresponded with the significantly higher CD8 + T-cell immunostaining that was orthogonally demonstrated amongst older patients (*P* < 0.01, Wilcoxon rank sum test) (Supplemental Data 9a-b).

We then interrogated whether tumor-intrinsic immunosuppressive pathways were enriched between the “cold” C1 cluster and the relatively “hot” C3 cluster. Among those enriched deregulated pathways identified were: PD1 signaling, CTLA4 pathway, IFNG signaling, and others (Fig. 3e). This suggests that C3 tumors are actively enhancing expression of signals that inhibit T-cell activation (PD-L1, CTLA4, IFNG) as well as molecules such as IDO1 that participate in the recruitment of immunosuppressive cells. Although TCR productive clonality measures were higher in C3 than in C1, they were still low overall (Supplemental Fig. 9i). The lack of high TCR productive clonality amongst C3 may be due to adaptive immune resistance mechanisms such as higher *PD-L1*, *CTLA4*, *IDO1* expression levels and the presence of myeloid-derived suppressor cells (MDSCs) in the C3 group as compared to C1 (Fig. 3e, f). For C1, the lack of TCR productive clonality could be explained in part by the lower levels of HLA antigen-presenting genes than seen in C3 (Supplemental Data 8e) and the number of deleted genes.

**Fig. 4:**
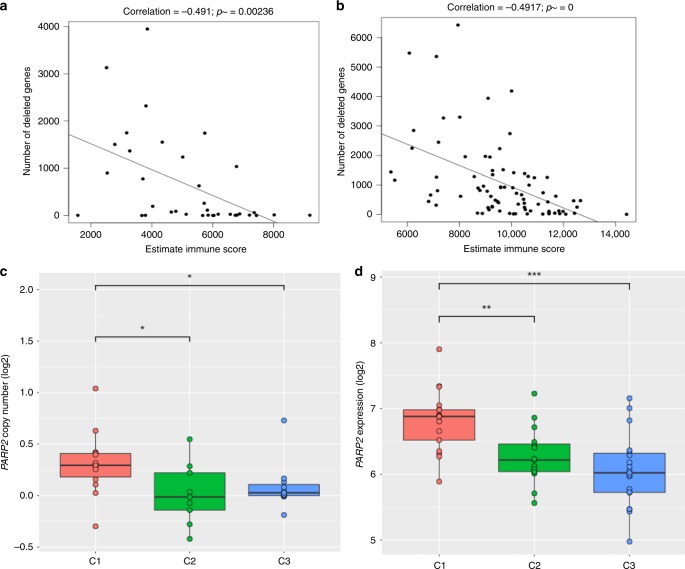
Copy number alterations and immune score associations. **a**, **b** Pearson correlation of the number of deleted genes and immune scores generated from ESTIMATE for our OS cohort (**a**) and the TARGET cohort (**b**). **c** Boxplot of *PARP2* copy number levels (in log2 level) in each of the immune infiltrate groups C1, C2, and C3 (as decribed in Fig. 3b). Wilcoxon rank sum test was used to determine significance. **d** Boxplot of *PARP2* gene expression levels (in log2 level) in each of the immune infiltrate groups C1, C2, and C3 (as decribed in Fig. 3b). Wilcoxon rank sum test was used to determine significance.

Recent studies have shown that high levels of genome aneuploidy in cancer are associated with lower levels of immune-related markers^30,31^. Indeed, higher number of copy number losses correlated with lower ESTIMATE immune infiltrate scores (*P* < 0.05, Pearson’s correlation), confirmed by the TARGET cohort (Fig. 4a, b). Also, specimens in C1 had a significantly higher number of deleted genes (Supplemental Fig. 10a). For the TARGET cohort, the number of genes with copy number gain were also negatively correlated with ESTIMATE immune scores whereas that was not seen in our cohort (Supplemental Fig. 11b). No significant relationship was observed among the three clusters in mutation burdens, numbers of predicted neoantigens, and numbers of rearrangements. A separate multiple regression analyses comparing genomic instability factors (number of losses, gains, rearrangements, somatic point mutations) and immune score revealed that losses had the greatest association with immune score (*P* < 0.01, multiple linear regression). To identify genes with genomic alterations that were significantly associated with immune infiltrate, we applied an integration analysis of genomic alterations and ESTIMATE immune infiltrate scores. As the top hit for copy number losses, both *TP53* loss and expression were significantly negatively correlated with ESTIMATE immune scores (See Methods and Supplemental Data 10b). Neither *PTEN* loss nor expression were associated (Supplemental Data 10a-c), contrary to what was found by Roh et al. in melanoma^31^. Thus, tumor type-specific associations may exist. Interestingly, the gene expression (Fig. 4c) and copy number gain of *PARP2* (Fig. 4d), a druggable target, are both significantly negatively associated with the ESTIMATE immune score. This relationship between *PARP2* expression and immune score was recapitulated in the TARGET dataset (Supplemental Fig. 11c). Thus, taken together, these data indicate that there are likely multiple immune-suppressive mechanisms in play in OS.

## Discussion

We conducted a comprehensive genomic and immune characterization of post-treatment primary, local recurrence, and metastasis OS specimens from a cohort of poor prognosis patients to determine molecular bases for the lack of response of OS to immune checkpoint therapy. We observed very few differences in the genomic landscapes and expression profiles across the three groups of samples. However, several interesting genomic features were identified in our cohort. First, the enrichment in focal clustered rearrangements amongst younger patients suggests that oncogenesis may be more driven by catastrophic chromothripsis events in young OS patients as compared to older adults. Second, our data also suggests that there is a temporal order to loss of *TP53* and subsequent ALT and WGD events to promote genome instability in OS. The role of ALT in genomic instability may provide a therapeutic target in OS, as proposed by Flynn et al.^32^. In addition, we also identified enrichment for mutation signatures 5 and 8 as Behjati et al.^6^, and found signature 8 is significantly associated with worse prognosis. Mutation signature 3 was the predominant mutational signature enriched in the TARGET cohort^33^. This discrepancy may be due to patient age (our cohort has more older patients) or that this enrichment is prevalent in particular subsets of OS that are yet to be defined.

We defined three broad immune subsets (C1, C2, C3) with low, intermediate, and high levels of immune infiltrate—so called “hot” and “cold” tumors. Although the level of immune infiltrate and activity is high in a subset of OS (C3), an ineffective immune response may be due to the lack of neoantigens or to the presence of tumor-intrinsic adaptive immune resistance mechanisms that allow for immune evasion or lack of T-cell activation (e.g., PD-L1, CTLA4, and MDSCs). Identification of a “hot” subset of OS supports the rationale for developing a biomarker-selected approaches to future immunotherapy trials in OS. Those with lower immune infiltrate were void of most types of immune cells and associated with higher number of genes with copy number loss as well as decreased HLA expression. This corroborates previous findings that copy number alterations and aneuploidy contribute to immune suppression^30,31^. In fact, out of multiple genomic alterations including copy number gains, copy number loss had the highest association with immune suppression in OS. This suggests copy number loss may have greater impact on cellular pathways than other alterations due to permanent loss of many genes^34^.

These data highlight the need to pursue multiple contributors to immune suppression in OS. It is unlikely that any single approach will be effective across this patient population. Future clinical and translational studies should (1) enrich for OS patients with high immune infiltrate/immune scores who may be more likely to benefit from immune checkpoint inhibitors with differing IO approaches for those with low vs. intermediate to high immune infiltrate, (2) explore novel approaches to enhance neoantigen expression such as radiation, NMD inhibition, or intratumoral vaccines, and (3) develop rational combinations of targeted therapy and immunotherapy. For example, amongst those with intermediate to high immune infiltrate (C2 and C3) strategies that combine ICI with other IO agents or targeted therapies to mitigate the immunosuppressive genes that were expressed at higher levels in these clusters such as TGFb or the PI3K pathway warrant further exploration. Conversely, PARP2 gains and increased expression were associated with low immune infiltrate in cluster group 1 (C1). Inhibiting PARP may render susceptibility to ICI, therefore this abundance of PARP supports the rationale for exploring combinations of PARP inhibitors and immunotherapy in OS. Ongoing translational studies from OS patients treated with immune checkpoint inhibitors may further inform the next steps in developing immunotherapy trials for this patient population^35^.

In conclusion, the immunogenomic landscape in OS is characterized by genomic complexity and significant disease heterogeneity. Rather than promoting a robust immune response, this genomic complexity may contribute to an immunosuppressive phenotype through multiple mechanisms that may present themselves as opportunities for novel therapeutic exploitation.

## Methods

### Patients and sample collection

Collection and use of patient samples were obtained by informed consent and approved by the University of Texas MD Anderson Cancer Center Institutional Review Board. We collected all available frozen OS specimens for immunogenomic characterization: frozen resected OS primary tumors (*n* = 14), locally recurrent OS tumors (*n* = 13), lung metastases (*n* = 27) and matched adjacent normal tissues as well as matched blood (*n* = 2) from 48 pediatric and adult patients with high-grade OS. The clinical patient characteristics are summarized in Table 1.

### Whole genome sequencing (WGS)

Genomic DNA (gDNA) was extracted with the QIAamp DNA Mini kit (Qiagen, Germantown, MD) and used for high depth paired-end whole genome sequencing. Whole genome sequencing data was generated at Baylor College of Medicine – Human Genome Sequencing Center (BCM-HGSC) using established library preparation and sequencing methods. Libraries were prepared using KAPA Hyper PCR-free library reagents (KK8505, KAPA Biosystems) on Beckman robotic workstations (Biomek FX and FXp models). Briefly, DNA (500 ng) was sheared into fragments of approximately 200-600 bp using the Covaris E210 system (96-well format, Covaris, Inc. Woburn, MA) followed by purification of the fragmented DNA using AMPure XP beads. A double size selection step was employed, with different ratios of AMPure XP beads, to select a narrow size band of sheared DNA molecules for library preparation. DNA end-repair and 3’-adenylation were then performed in the same reaction followed by ligation of the barcoded adaptors to create PCR-Free libraries, and the library run on the Fragment Analyzer (Advanced Analytical Technologies, Ames, Iowa) to assess library size and presence of remaining adapter dimers. This was followed by qPCR assay using KAPA Library Quantification Kit using their SYBR® FAST qPCR Master Mix to estimate the size and quantification. WGS libraries were sequenced on the Illumina HiSeq-X instrument using the Reagent Kit v2.5 (FC-501-2501) and libraries were loaded at an average concentration of 280 pM to generate 150 bp paired-end reads. Unique aligned sequence in these samples varied between 209 Gb - 255 Gb per sample. The average insert sizes in these samples were 435 bp median and 414 bp mode. For each sample, the reads were mapped to the hg19 reference genome using BWA-MEM, followed by the downstream analyses detailed below.

### Somatic point/indel mutation calling and related analysis

Somatic point variants were called from aligned WGS data using MuTect^36^. High quality variants were defined as those with a minimum tumor read depth of ≥30, minimum matched normal read depth of ≥15, and minimum alternate allele frequencies in the tumor and normal as ≥0.05 and ≤0.01, respectively. Kataegis were identified as those genomic regions containing eight or more consecutive mutations with an average intermutation distance of less than or equal to 1000 bp using the R package, ClusteredMutations^37^. Indel variants were called using Pindel^38^. Pindel raw calls were further filtered to select for calls with score >30, ESP6500 and 1000 G population minor allele frequencies >0.01, and not intronic. Neoantigens were predicted from the point and indel mutations using PHLAT^39^ and NetMHC^40^.

We used two methods to identify mutation signatures in our samples. First, we used Nonnegative Matrix Factorization (NMF) to do de-novo mutation signatures discovery from our patient samples based on the method described by Alexandrov et al.^41^. Then, we compared the two identified mutation signatures with the 30 published COSMIC mutation signatures (http://cancer.sanger.ac.uk/cosmic/signatures) using the cosine similarity metric. Second, we factorized the mutation spectrum based on the 30 known COSMIC mutation signatures using the fast combinatorial non-Negative least-square algorithm^42^ to solve the NMF problem.

### Somatic copy number calling and related analysis

Total copy number calls were derived using HMMcopy^43^, and log2 scores >0.5 were considered gains while log2 scores <−0.5 were considered losses. The weighted genome instability index (WGII)^44^ was used to quantitatively characterize portion of the genome with copy number alterations as a measure of chromosomal instability. Focal recurrent copy number alterations were identified using GISTIC 2.0^45^ at 95% confidence level. The R package, CNTools^46^, was used to process the segmented total copy number data into gene-level data. Allele specific copy number calls, purities, ploidies, and loss of heterozygosity of the tumor samples were derived using Sequenza^47^ with default parameters. Chromothripsis was detected using total copy number profiles by CTLPScanner^48^.

### Genome doubling

An in-house algorithm was developed with modifications based on previously published studies^17,44^ was applied to detect genome doubling in the samples. The algorithm is detailed below.

(1) Summarize absolute copy number of two alleles in chromosome arm level.

(2) Calculate total number of aberrations (relative to diploid) for each sample (*N*_*s*_)

$N_s = \mathop {\sum }\limits_{i = 1}^{44} \mathop {\sum }\limits_{j = 1}^2 \left| {{\mathrm{CNV}}_{i,j} - 1} \right|$where CNV_*i,j*_ is absolute copy number in arm *i* and *j* allele for sample *s*. We only consider 22 chromosomes (44 arms).

Calculate probability of gain (*N*_gain_) and loss (*N*_loss_) for sample *s*.

$$P_{s,{\mathrm{gain}}} = N_{{\mathrm{gain}}}/N_s$$

$$P_{s,{\mathrm{loss}}} = N_{{\mathrm{loss}}}/N_s$$

(4) Do 10,000 simulation. In each simulation for a sample, we start with 1 copy in each allele and each arm. Then we sample *N*_*s*_ gain and loss based the gain and loss probabilities, and add the gain or loss to a randomly selected allele and arm.

(5) Calculate number of simulations (M) in which number of arms with the major allele copy number ≥2 were higher than that observed in the sample. A *p*-value for genome doubling of a sample $$= {\raise0.5ex\hbox{$\scriptstyle M$}\kern-0.1em/\kern-0.15em \lower0.25ex\hbox{$\scriptstyle {10,000}$}}$$.

(6) A *p*-value threshold of 0.001 was used to determine if a sample went through genome doubling.

(7) Samples with *p* < 0.001 and major copy number = 2 have 1 genome doubling. Samples with *p* < 0.001 and major copy number ≥3 have >1 genome doubling.

### Rearrangement calling and rearrangement signatures

Structural rearrangements were detected from aligned WGS data using BReakpoint AnalySiS (BRASS)^13^. We used the list of fragile site genomic locations from Fungtammasan et al.^49^ to intersect with the Brass calls to identify rearrangements in the fragile sites. Expressed rearrangements identification is described in the RNA sequencing section.

The rearrangement signatures in the samples were identified using the strategy revised from the previously published studies^13^ to identify rearrangement signatures in the samples. First, we separated rearrangements that occurred as focal catastrophic events (clustered) or focal driver amplicons (unclustered). Nik-Zainal et al.^13^ used the piecewise constant fitting method to determine clustered breakpoints in each sample if the mean of inter-breakpoint distance of clustered regions are at least 10 times smaller than average inter-breakpoint distance of the whole genome. This method will be biased if majority of rearrangements are resulted from chromthripsis. Therefore, we identified clustered rearrangement breakpoints that locate in the chromothripsis regions using CTLPScanner^48^. Second, clustered and unclustered rearrangements are subclassified into deletions, inversions, tandem duplications, and interchromosomal translocations. Third, except interchromosomal translocations, other clustered and unclustered rearrangements are further categorized by their lengths (1–10 kb, 10 kb–100 kb, 100 kb–1 Mb, 1 Mb–10 Mb, more than 10 Mb). Finally, we applied Nonnegative Matrix Factorization (NMF) to the classified matrix of 32 categories of rearrangements to do de-novo rearrangement signatures discovery from our samples. The silhouette width-based method determined two optimal signatures. Comparisons the two identified rearrangement signatures with the 6 published signatures identified in the 560 breast cancer samples using the cosine similarity metric found the two signatures mostly correlate with the signature 2 (correlation = 0.94) and 4 (correlation = 0.88), respectively.

### Telomere length

Telomere lengths of tumor and normal samples were estimated from WGS data using the TelSeq^50^. The tool estimates telomere length of a sample by counting the number of reads containing telomeric repeats, TTAGGG. Considering telomere length may be associated with patient age or other individual factor, we used ratio of telomere lengths between pairs of tumor and normal samples (T/N TL ratio) in our analysis.

### Suclonal architecture

#### Consensus mutations

We used MuSE^51^ to call the somatic mutations based on paired tumor normal paired DNAseq data. The SNV called by MuSE are compared to MuTect. If a mutation is called by both variant caller, we define it as a consensus mutations. The we define a statistic, ${\mathrm{overlap}}\,{\mathrm{ratio}} = \frac{{{\mathrm{number}}\,{\mathrm{of}}\,{\mathrm{consensus}}\,{\mathrm{mutations}}}}{{{\mathrm{number}}\,{\mathrm{of}}\,{\mathrm{MuTect}}\,{\mathrm{mutations}}}}$, to measure the agreement between the two callers. A higher overlap ratio indicates a better agreement between the two callers. Supplemental Figure 12a shows a summary histogram of overlap ratio of the two callers.

There is high correlation between tumor purity and consensus calls (Supplemental Figure 12b) as well as the mutation number versus the overlap ratio (Supplemental Figure 12c).

#### Subclonal mutation reconstruction

We applied CliP (Clonal structure identification through pair-wise penalization)^52^, for subclonal mutation architecture reconstruction, using the consensus mutations. Subclonal architecture reconstruction groups the somatic SNVs according to the proportion of cells in a given sample that harbor these SNVs (i.e., the Cellular Prevalences of SNVs). CliP utilizes a model based clustering approach and estimates the model parameters using a regularized maximum likelihood estimation implemented using alternating direction method of multipliers (ADMM). CliP is much faster than most existing methods, and has comparable/better performance which makes CliP ultimately suitable for large studies. Importantly. Furthermore, CliP does not require the number of clusters as input, which was required by most clustering based methods. Sequenza estimated tumor purity and copy number variant data of the 35 OS samples. Supplemental Fig. 12d shows the summary of subclonal structure of the 35 samples.

#### RNA sequencing and related analysis

Total RNA of samples was extracted and libraries made from cDNA using the NuGEN Ovation Ultralow Library System V2 (San Carlos, CA). For estimation of expressed neoantigens and immune therapy samples, libraries were generated using Agilent SureSelect^XT^ RNA Direct (Agilent Technologies) (Supplemental Data 11). RNA sequencing reads of the samples were mapped to the hg19 reference genome using the STAR aligner^53^. For calculation of gene expression, raw count data of each gene were first obtained using with HTSeq^54^ and are normalized by scaling the raw library size using calcNormFactors in edgeR package in R^55^. Then, Voom transformation was applied to normalized counts and a linear model fit to the data for differential expression analysis using the Limma package^56^. Fusion transcripts were detected from RNASeq data using MapSplice^57^. To identified expressed rearrangements, we also integrated in-frame rearrangements and fusion transcripts to identify expressed rearrangements in each sample, whose genomic breakpoints detected from WGS data and fusion transcript junction regions detected from RNA-seq are in the same genic regions.

Immune infiltration scores were calculated from the gene expression data using several methods: ESTIMATE^22^, TIMER^28^, and cytolytic scores (calculated as the geometric mean of *GZMA* and *PRF1* gene expression values)^29^. Immune infiltrate profiles of samples were generated using single sample Gene Set Enrichment Analysis (ssGSEA) enrichment scores of each immune gene list from Charoentong et al.^27^. Pathway analyses of differentially expressed genes between any two immune clusters were performed using Gene Set Enrichment Analysis (GSEA)^58^.

#### Genetic alterations associated with immune infiltrates

We used an integrative analysis to identity genetic alterations that are associated with immune infiltrates. We did correlation analysis of the ESTIMATE immune score respectively with gene alterations and gene expression. The product truncated method was then applied to combine *p*-values of two correlation analyses for each gene. Genes with significant combined *p*-values (*p* < 0.01) would have significant correlations of the immune score both with its genetic alterations and gene expression. We identified *PARP2* whose amplifications and gene expression are both significantly negatively correlated with the ESTIMATE immune score.

#### Reverse phase protein array

Tumor lysates were serially diluted from undiluted to 1:16 and arrayed on nitrocellulose-coated slides in an 11 × 11 format and probed with 304 unique antibodies (Supplemental Data 12a). Of those 299 antibodies passed quality assurances and were used for further analysis. Following antibody probing of lysates on the slide, a flatbed scanner was used to produce 16-bit tiff image with subsequent spot identification and density quantification using the Array-Pro Analyzer (Meyer Instruments). SuperCurve Rx64 3.1.1^59^ was used to derive normalized log2 values with further normalization for protein loading and transformation into linear values. We only included the data for the **i**ndividual antibodies with QC Scores higher than 0.80 in the heatmap. The heatmap was generated in Cluster 3.0^60^ as a hierarchical cluster using Pearson Correlation and a center metric. The resulting heatmap was visualized in Treeview^61^. Pathway analysis was conducted as previously described. A listing of the antibodies used for each pathway analyzed is in Supplemental Data 12b).

#### T-cell receptor sequencing

Immunosequencing of the CDR3 regions of human TCRβ chains was performed using the ImmunoSEQ Assay (Adaptive Biotechnologies). Through the ImmunoSEQ Analyzer platform, TCRB rearrangements were extracted along with their read counts, read proportions and appropriate TCRB gene usage. T-cell clonality was defined as 1-Peilou’s evenness and was calculated on productive rearrangements by:

$$1 + \left( {\mathop {\sum }\limits_i^N p_i\log _2p_i/\log _2\left( N \right)} \right)$$

where _*pi*_ is the proportional abundance of rearrangement *i*, and *N* is the total number of rearrangements. Difference in T-cell density, unique rearrangements, and clonality within samples were calculated as the difference between the highest and lowest values in a tumor, expressed as a percentage of the highest value [(max–min)/max × 100]. MOI is a measure of the similarity in the T-cell repertoire between samples ranging from 0 to 1, taking into account the specific rearrangements and their respective frequencies, with an MOI of 1 being an identical T-cell repertoire. Enriched T-cell rearrangements were calculated by comparing the peripheral T-cell repertoire in patients with available blood to the tumor T-cell repertoire. Subsequent analyses were performed only on significantly enriched T-cell rearrangements within the tumor. Next, the data was split based on the rearrangements observed in each sample and the proportion of the most abundant clonotypes were calculated. Following which, clonal space homeostasis was modeled as an extension of population dynamics. A ratio of 0 to 1 was used to determine how much space was occupied by each TCR clonotype. Clonal spaces were broken down in Fig. 4b as rare (0 < X < 1e-5), small (1e-5 < X < 1e-4), medium (1e-4 < X 1e-3), large (0.001 < X < 0.01) and hyper expanded (0.01 < X ≤ 1) clones.

#### Immunohistochemistry

Decalcified formalin-fixed, paraffin-embedded (FFPE) blocks of osteosarcoma tissue from surgical resection specimens were retrieved from MD Anderson’s institutional tumor bank. All specimens were fixed for at least 8 h in buffered formalin-fixed and decalcified using 10% formic acid. Slides of hematoxylin and eosin–stained sections were reviewed with a bone and soft pathologists and two tissue cores (0.6-mm diameter) were extracted from representative tumor areas of the FFPE to construct tissue microarrays. Slides of 4-µm-thick unstained tissue sections were prepared from the tissue microarrays of decalcified FFPE human OS specimens retrieved from the UT MD Anderson’s institutional tumor bank. Immunohistochemical staining was independently scored by two pathologists who were blinded to the clinical data at the time of assessment. The list of antibodies and their dilutions are shown in the Supplemental Data 13. The correlation between the continuous biomarkers and age at diagnosis was measured by Spearman correlation coefficient. Wilcoxon rank sum test was used to compare age at diagnosis between two biomarker subgroups.

#### Data analysis of the TARGET project

Copy number segment and gene expression row count data of TARGET OS samples were downloaded from TARGET data matrix (https://ocg.cancer.gov/programs/target/data-matrix). The same methods used in our cohort samples were applied to process and analyze these data.

### Reporting summary

Further information on research design is available in the Nature Research Reporting Summary linked to this article.

## Supplementary information

Description of Additional Supplementary Files

Supplementary Data 1

Supplementary Data 2

Supplementary Data 3a

Supplementary Data 3b

Supplementary Data 4a

Supplementary Data 4b

Supplementary Data 5

Supplementary Data 6

Supplementary Data 7

Supplementary Data 8a

Supplementary Data 8b

Supplementary Data 8c

Supplementary Data 8d

Supplementary Data 8e

Supplementary Data 9a

Supplementary Data 9b

Supplementary Data 10a

Supplementary Data 10b

Supplementary Data 10c

Supplementary Data 11

Supplementary Data 12a

Supplementary Data 12b

Supplementary Data 13

Supplementary Information

Reporting Summary

## Data Availability

The whole genome and RNA sequencing data will be at the European Genome-phenome Archive under the study accession number: EGAS00001003247. The RPPA data is found under the study accession number: TCPA00000004. T-cell receptor sequencing is housed at Adaptive Technologies: [10.21417/CW2020NC].
