# Supplementary material for: Immuno-genomic landscape of osteosarcoma
Source: Nat Commun. 2020 Feb 21;11:1008. doi: 10.1038/s41467-020-14646-w (PMC7035358; doi:10.1038/s41467-020-14646-w)
Supplement: Supplementary file 1 — Description of Additional Supplementary Files [file 41467_2020_14646_MOESM1_ESM.pdf]

Description of Additional Supplementary Files

Supplemental Data 1: Clinical characteristics and assay tally
Supplemental Data 2: WGS coverage
Supplemental Data 3a: Genes with functional mutations in at least 6 specimens
Supplemental Data 3b: Recurrent point mutations in at least 6 specimens
Supplemental Data 4a: Pathways enriched in primary versus lung metastases specimens
Supplemental Data 4b: Pathways enriched in lung metastases as compared to primary specimens
Supplemental Data 5: Rearrangements within fragile sites
Supplemental Data 6: Telomere length correlation against the expression of 70 genes involved in telomere maintenance (Nabetani and Ishikawa, 2011)[19]. Only significant results are shown.
Supplemental Data 7: Correlation between tumor necrosis and genomic features. Only significant results are shown.
Supplemental Data 8a: Immunohistochemistry categorical biomarkers factors
Supplemental Data 8b: Proportion of CD3 core density subgroups among patients with lung/distant tumor and primary tumor
Supplemental Data 8c: Comparison of biomarkers between patients with lung/distant tumor and primary tumor
Supplemental Data 8d: T-cell receptor productive clonality
Supplemental Data 8e: Gene expression levels of antigen presenting complex members
Supplemental Data 9a: Comparison of age at diagnosis between two CD8 density subgroups among the patients with localized stage at initial diagnosis
Supplemental Data 9b: Immunohistochemistry correlation between biomarkers and age at diagnosis
Supplemental Data 10a: Significant associations between genomic alterations and ESTIMATE immune score
Supplemental Data 10b: Significantly associations between copy number alteration, gene expression and ESTIMATE immune score association
Supplemental Data 10c: Significantly associations between copy number alteration, gene expression and immune score association (Roh et al., 2017)31
Supplemental Data 11: RNASeq coverage
Supplemental Data 12a: RPPA antibodies
Supplemental Data 12b: RPPA proteins used for developing pathway scores
Supplemental Data 13: Antibody information and dilution schemes for immunohistochemical stains
