# Supplementary material for: Immuno-genomic landscape of osteosarcoma
Source: Nat Commun. 2020 Feb 21;11:1008. doi: 10.1038/s41467-020-14646-w (PMC7035358; doi:10.1038/s41467-020-14646-w)
Supplement: Supplementary file 25 — Supplementary Information [file 41467_2020_14646_MOESM25_ESM.pdf]

Supplemental Figure 1

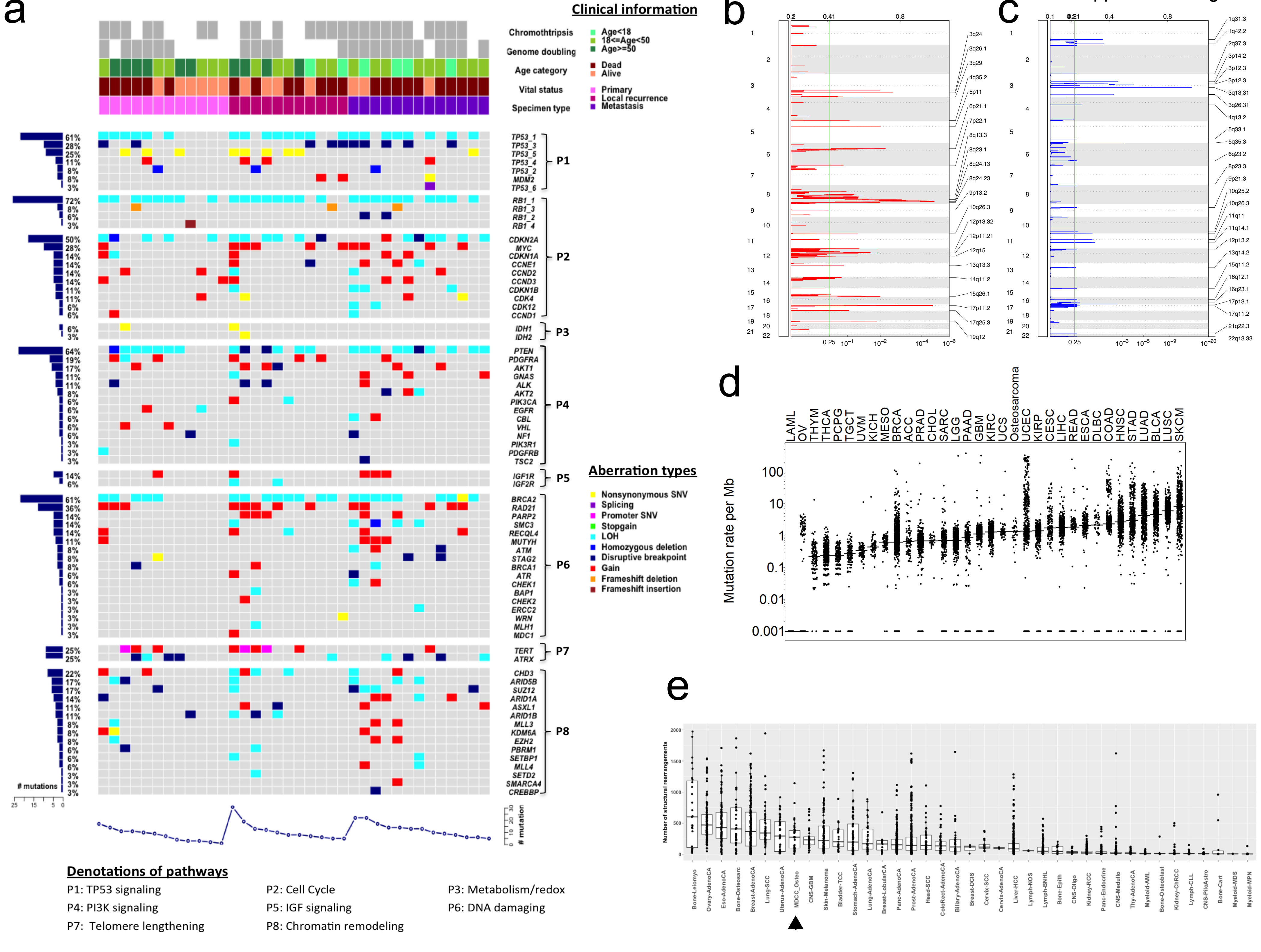

**Supplemental Figure 1.** Genomic Landscape **a.** Alteration landscape of driver genes from Behjati et al., 2017 and those mentioned in the text (TERT, ATRX and PARP2): their point mutations, copy number alterations, disruptive breakpoints, losses of heterozygosity, age category, vital status, specimen types, as well as occurrences of chromothripsis and whole genome doubling (WGD) (gray = chromothripsis/WGD is present, white = chromothripsis/ WGD is absent). **b.-c.** GISTIC plots of recurrent focal copy number gains (**b.**) and losses (**c.**) across our cohort. GISTIC q-values (x-axis) for copy number gains and losses are plotted across the genome (y-axis). **d.** Point mutation rate per megabase in our OS specimens as compared to TCGA cancer types. The results shown here are in part based upon data generated by the TCGA Research Network: http://cancergenome.nih.gov/. **e.** Number of rearrangements as called by BRASS in our cohort (MDACC_Osteo) as compared to ICGC tumor types. The results show here are in part based upon data generated by ICGC. http:// www.icgc.org/.

Supplemental Figure 2

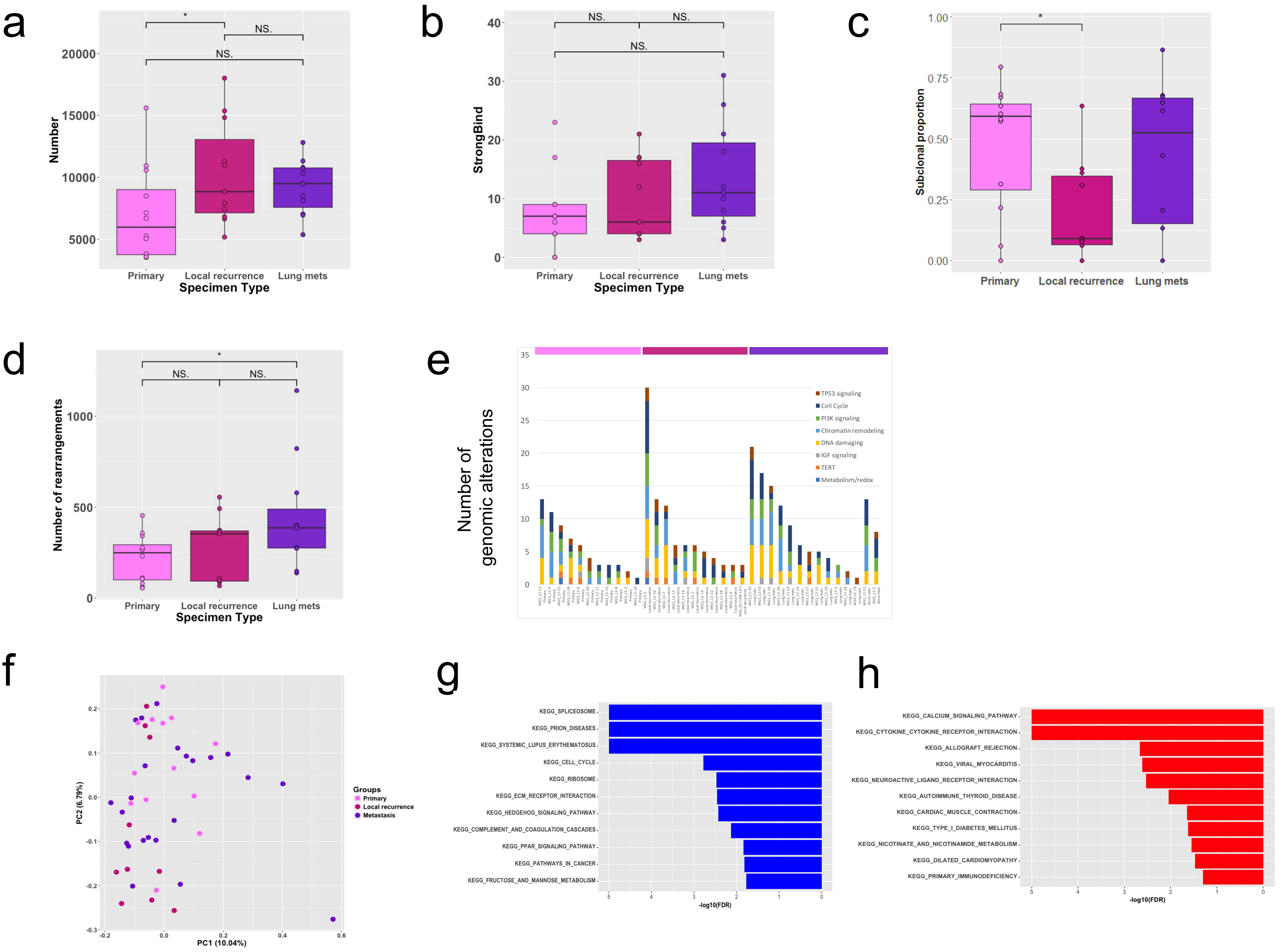

**Supplemental Figure 2.** Comparisons across tumor specimen type (primary, local recurrence and lung metastases). Pairwise *t*-test was used to determine significance. **a.-b.** Boxplot showing the total number of somatic mutations (**a.**), predicted strong-binding neoantigens (**b.**), proportion of mutations that are subclonal (**c.**), and rearrangements identified by BRASS according to tumor specimen type (**d.**). **e.** Stacked bar plot showing the tallies of genomic alterations of selected pathways in Supplemental Fig. 1a. **f.** Principal component analysis of gene expression data. **g.-h.** Top KEGG pathways significantly up-regulated in (**g.**) primary samples (**h.**) lung metastases, by GSEA analysis.

Fig.S3

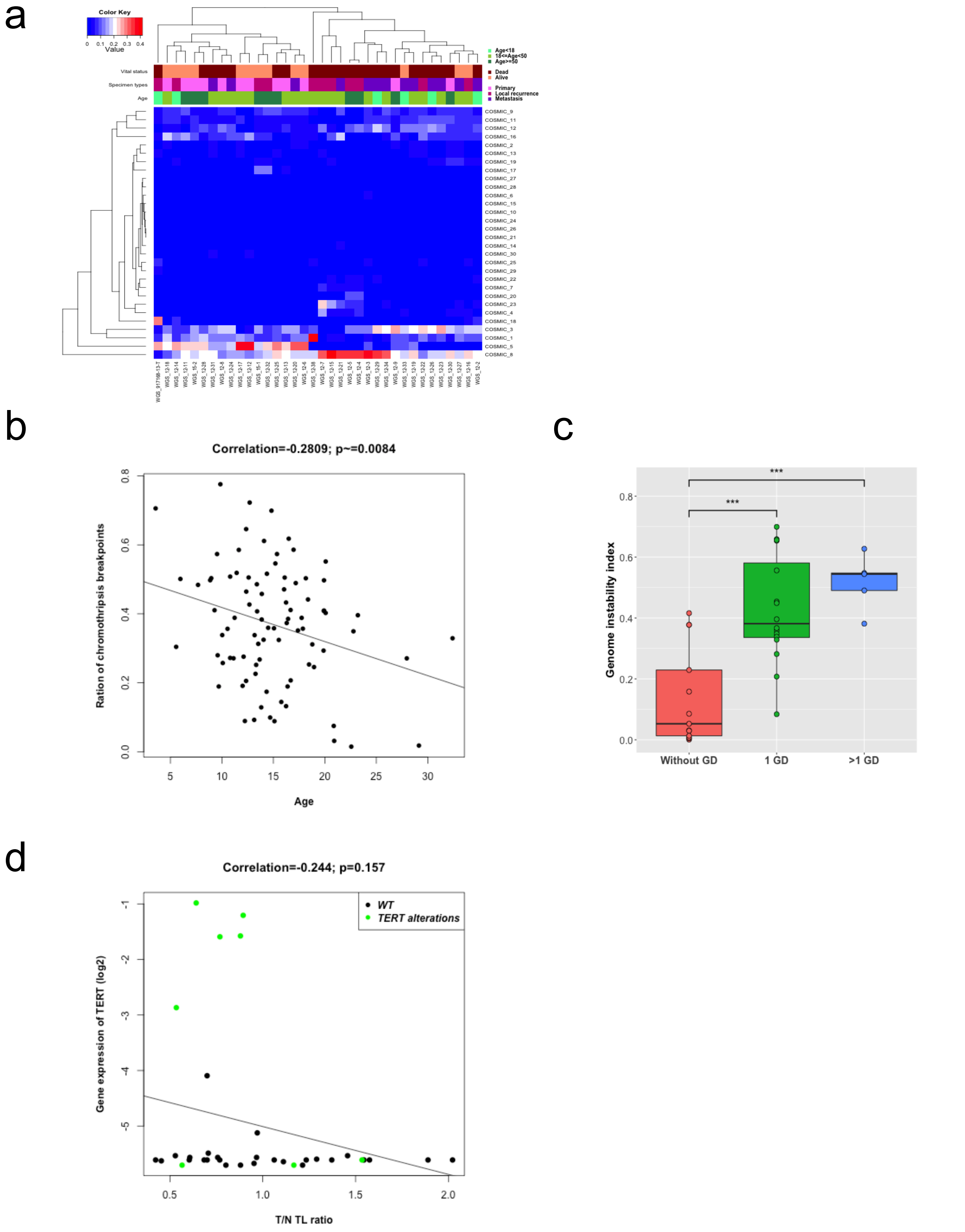
a
Color Key
Value
Vital status
Specimen types
Age
Age<18
18<=Age<50
Age>=50
Dead
Alive
Primary
Local recurrence
Metastasis
COSMIC_9
COSMIC_11
COSMIC_12
COSMIC_16
COSMIC_2
COSMIC_13
COSMIC_19
COSMIC_17
COSMIC_27
COSMIC_28
COSMIC_6
COSMIC_15
COSMIC_10
COSMIC_24
COSMIC_26
COSMIC_21
COSMIC_14
COSMIC_30
COSMIC_25
COSMIC_29
COSMIC_22
COSMIC_7
COSMIC_20
COSMIC_23
COSMIC_4
COSMIC_18
COSMIC_3
COSMIC_1
COSMIC_5
COSMIC_8
b
Correlation=-0.2809; p~=0.0084
Ration of chromothripsis breakpoints
Age
c
Genome instability index
Without GD
1 GD
>1 GD
***
d
Correlation=-0.244; p=0.157
WT
TERT alterations
Gene expression of TERT (log2)
T/N TL ratio

**Supplemental Figure 3.** Genomic Features. **a.** Unsupervised hierarchical clustering heatmap of mutation signature scores (level is depicted according to the color scale) across the cohort samples with clinical annotations. **b.** Spearman correlation of the ratio of breakpoints associated with chromothripsis against age of diagnosis for the TARGET cohort. **c.** Boxplot showing the genome instability index (see Methods) for specimens without genome doubling (GD), genome doubling that occurred once, and genome doubling occurring more than once. The significances of the comparisons were from the Wilcoxon rank sum test. **d.** Pearson correlation of normalized telomere length (x-axis) and *TERT* gene expression level (in log2 scale) of samples (y-axis). Samples with *TERT* alterations were marked as green color.

Supplemental Figure 4

a

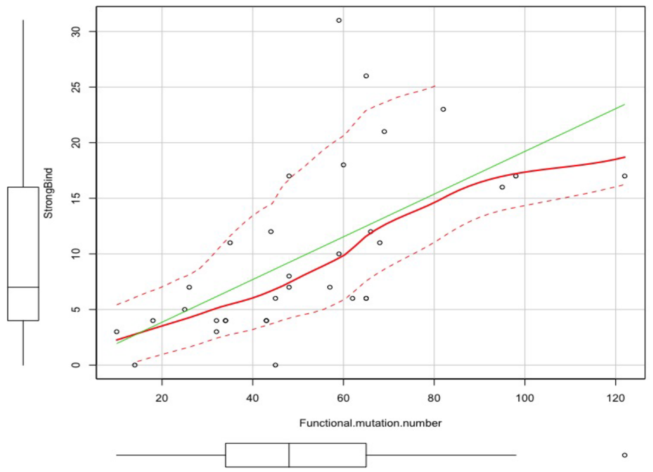


b

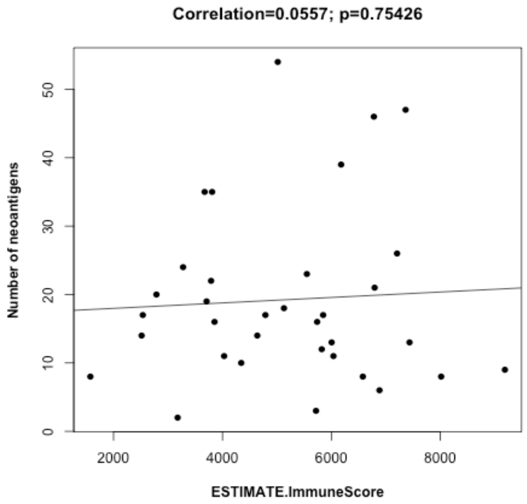


c

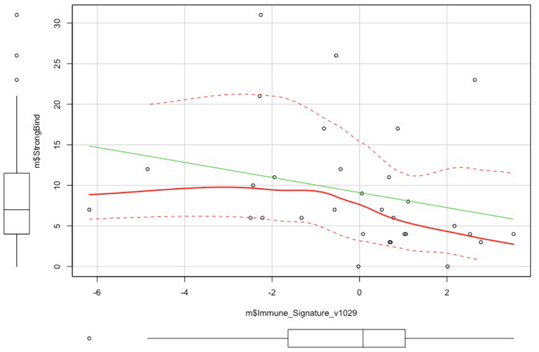


d

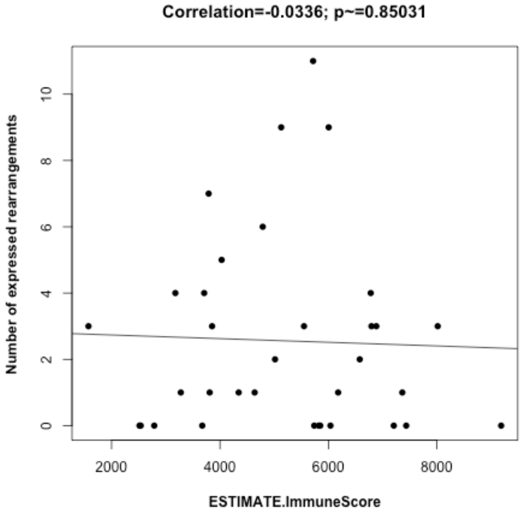

**Supplemental Figure 4.** Somatic alterations and immune infiltrate **a.** Regression analysis of functional mutation burden as positively associated with predicted neoantigens
(StrongBind). **b.** Pearson correlation of neoantigen burden and ESTIMATE immune score. **c.** Regression analysis of RPPA Immune signature (see Supplemental Methods and Table S11B) and the number of predicted antigens (StrongBind). **d.** Perason correlation of somatic rearrangements (expressed rearrangements) found in both WGS and RNASeq and ESTIMATE immune score.

a

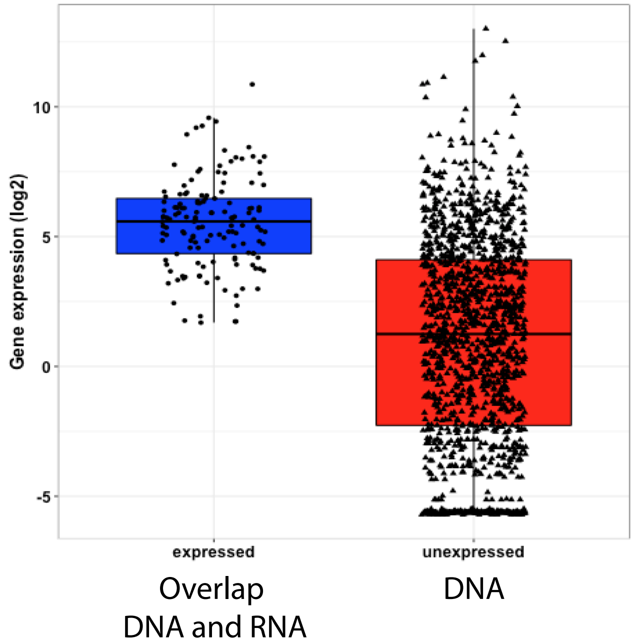
10
5
0
-5
Gene expression (log2)
expressed
unexpressed
Overlap
DNA and RNA
DNA

b

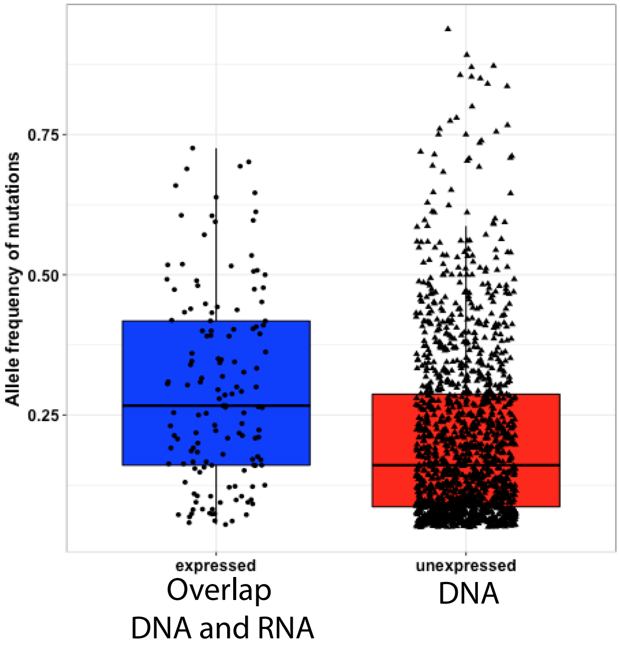
0.75
0.50
0.25
Allele frequency of mutations
expressed
unexpressed
Overlap
DNA and RNA
DNA

**Supplemental Figure 5.** Expression of somatic mutations **a.** Gene expression levels of mutations found in WGS and not RNASeq (unexpressed) or overlapping in both DNA and RNA sequencing (expressed) calls. **b.** Variant allele frequencies of mutations found in WGS alone (unexpressed) or in both DNA and RNA sequencing (expressed) calls.

Supplemental Figure 6

a

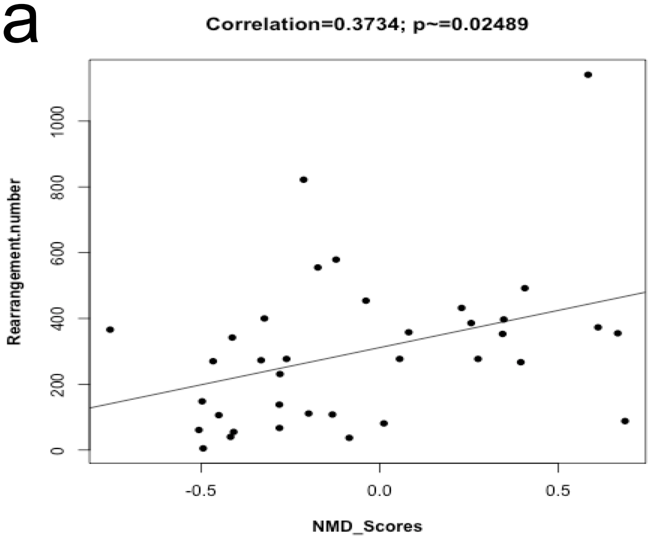


b

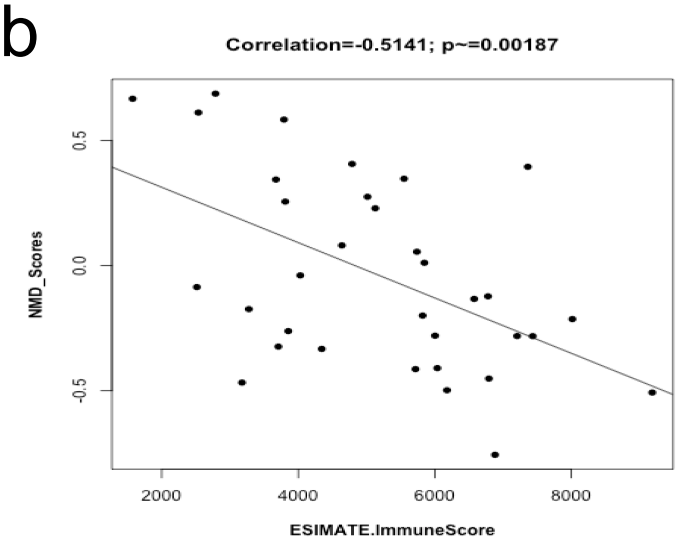

**Supplemental Figure 6.** Nonsense mediated decay. **a.** Pearson correlation of total number of rearrangements and the score of nonsense mediated decay pathway signature (NMD_Scores). **b.** Pearson correlation of the ESTIMATE immune score and the score of nonsense mediated decay pathway signature.

# Supplemental Figure 7

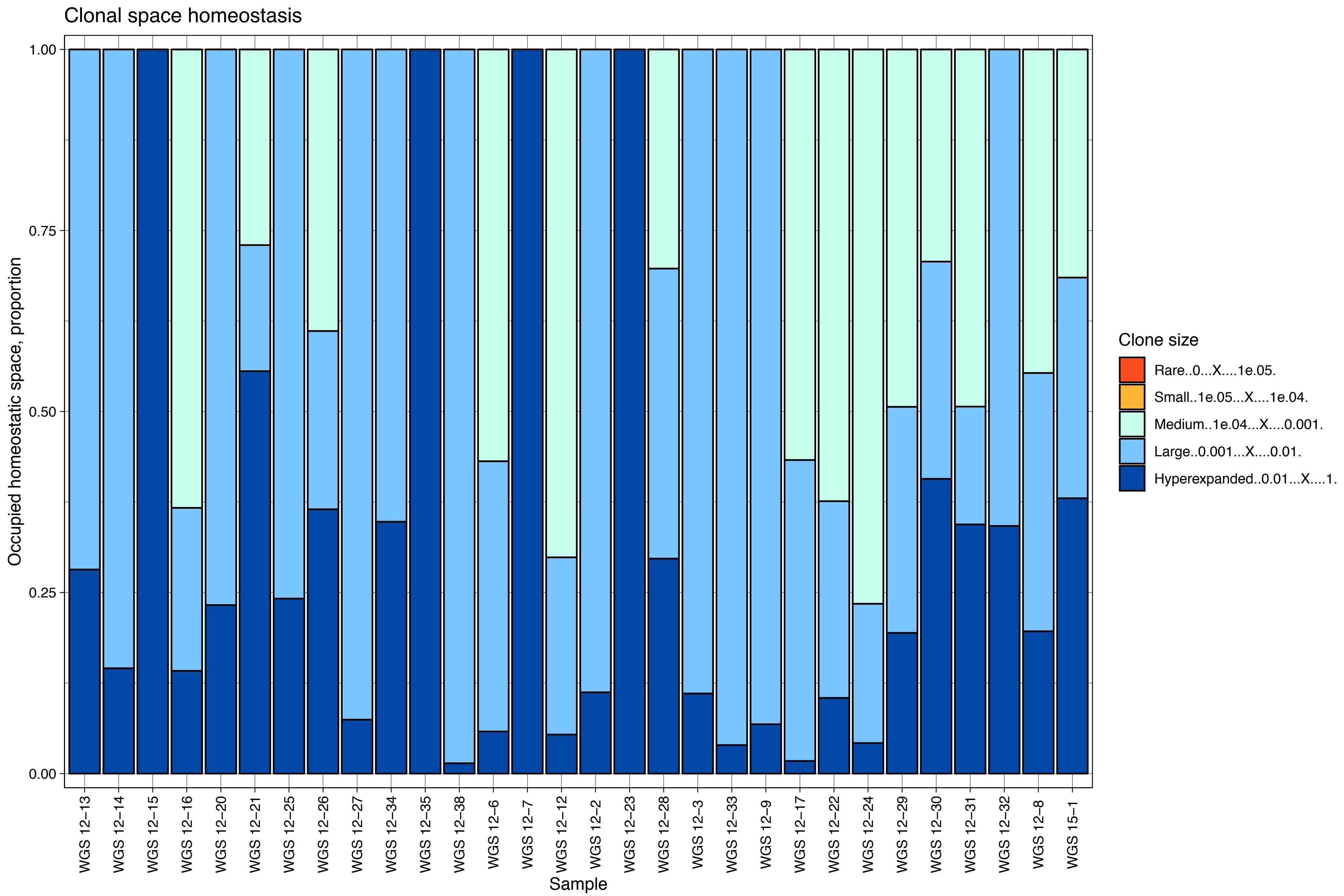

**Supplemental Figure 7.** Stacked bar plot showing the occupied homeostatic space of the T-cell clones. Each T-cell clone makes up a particular proportion of the entire T-cell clone population. The proportions are binned into categories based on proportion ranges. T-cells that make up 0 to 1e-05 proportion of the entire T-cell clonal population are placed into the bin entitled, “Rare”. The ranges of the other proportions are indicated: Small (range 1e-05 – 1e-04), Medium (range 1e-04 – 0.001), Large (range 0.001-0.01), and Hyperexpanded (range 0.01-1).

Supplemental Figure 8

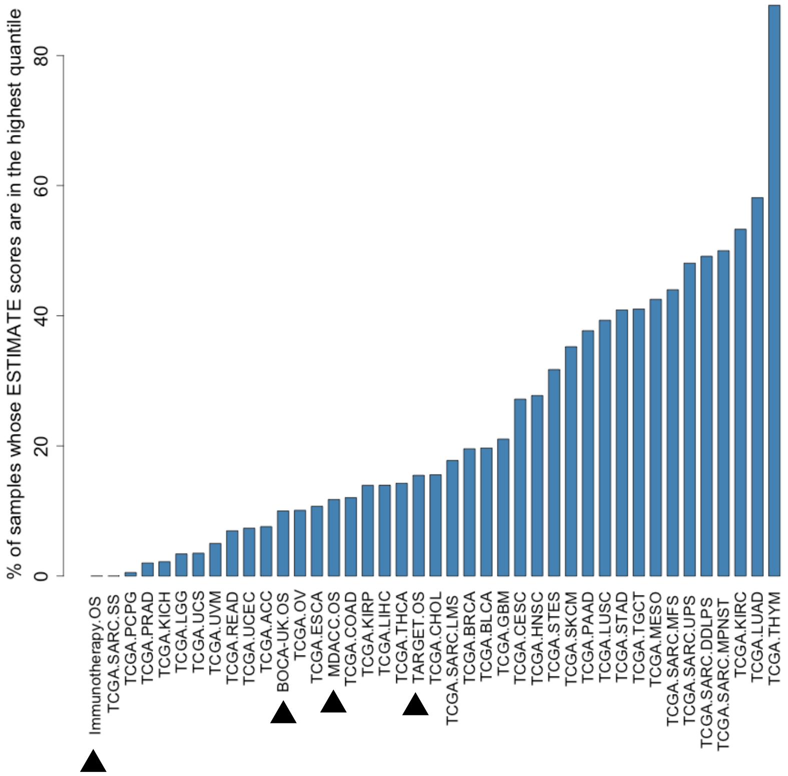

**Supplemental Figure 8.** Barplot of pan-cancer ESTIMATE scores. Barplot of the percentage of samples whose ESTIMATE immune scores are within the top quartile (y-axis) of ESTIMATE immune scores across all TCGA and OS cohorts examined (MDACC, BOCA-UK (ICGC), TARGET, immune checkpoint therapy) (x-axis).

Supplemental Figure 9

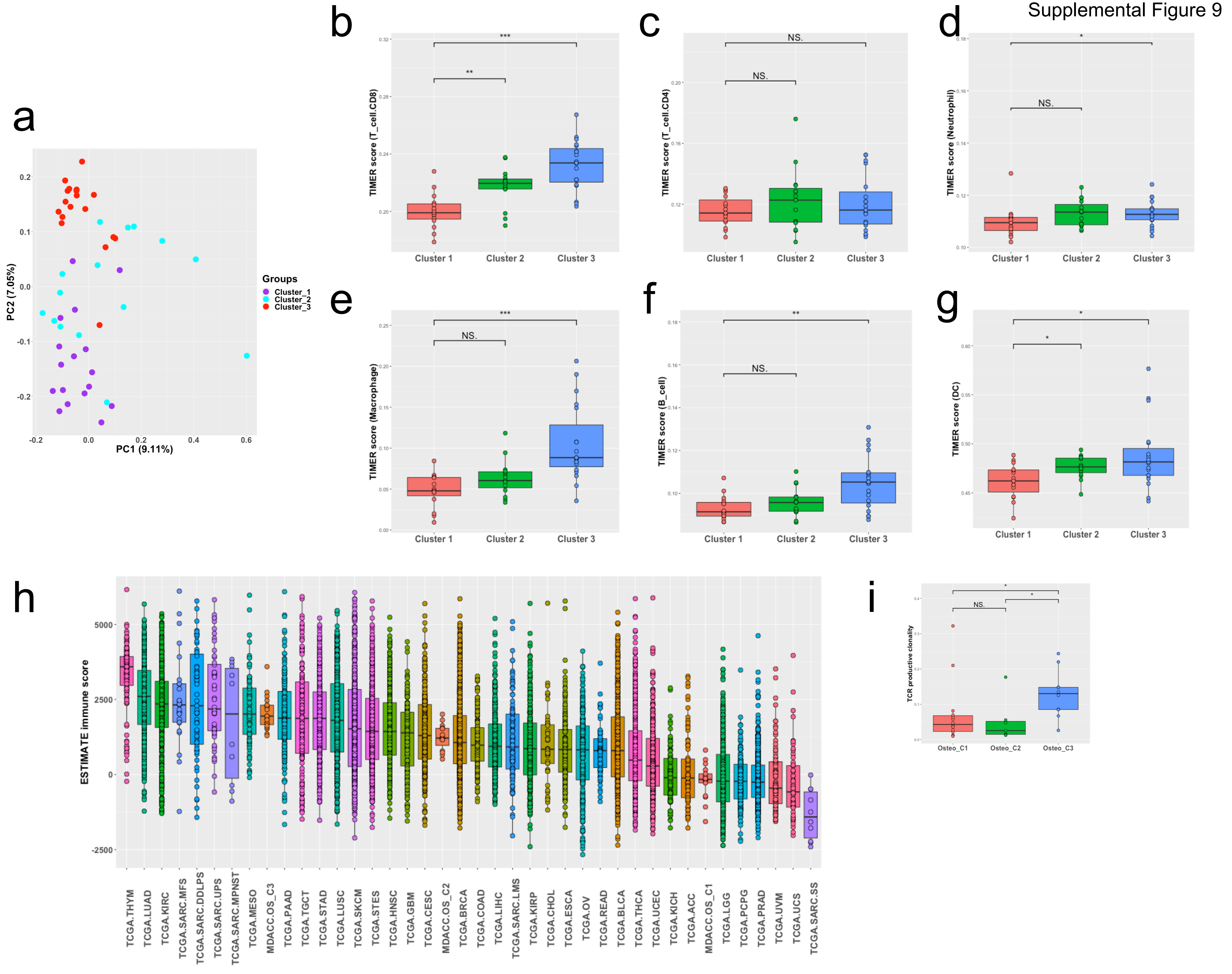

**Supplemental Figure 9.** Immune clusters (as described in Fig. 3b) **a.** Principal component plot of the immune clusters identified in hierarchical clustering from Fig. 3b. **b.-g.** Boxplots comparing immune cell type-specific scores based on TIMER for immune clusters 1 to 3 (**b.** CD8+ T-cells; **c.** CD4+ T-cells; **d.** neutrophils; **e.** macrophages; **f.** B-cells; **g.** dendritic cells). The Wilcoxon rank sum test was used to determine significance of the comparisons. **h.** Boxplot of ESTIMATE immune scores displaying at the three immune clusters (C1, C2, and C3) distribute against TCGA samples) **i.** T-cell receptor (TCR) productive clonality across the three clusters C1, C2, and C3. The Wilcoxon rank sum test was used to determine significance of the comparison.

Supplemental Figure 10

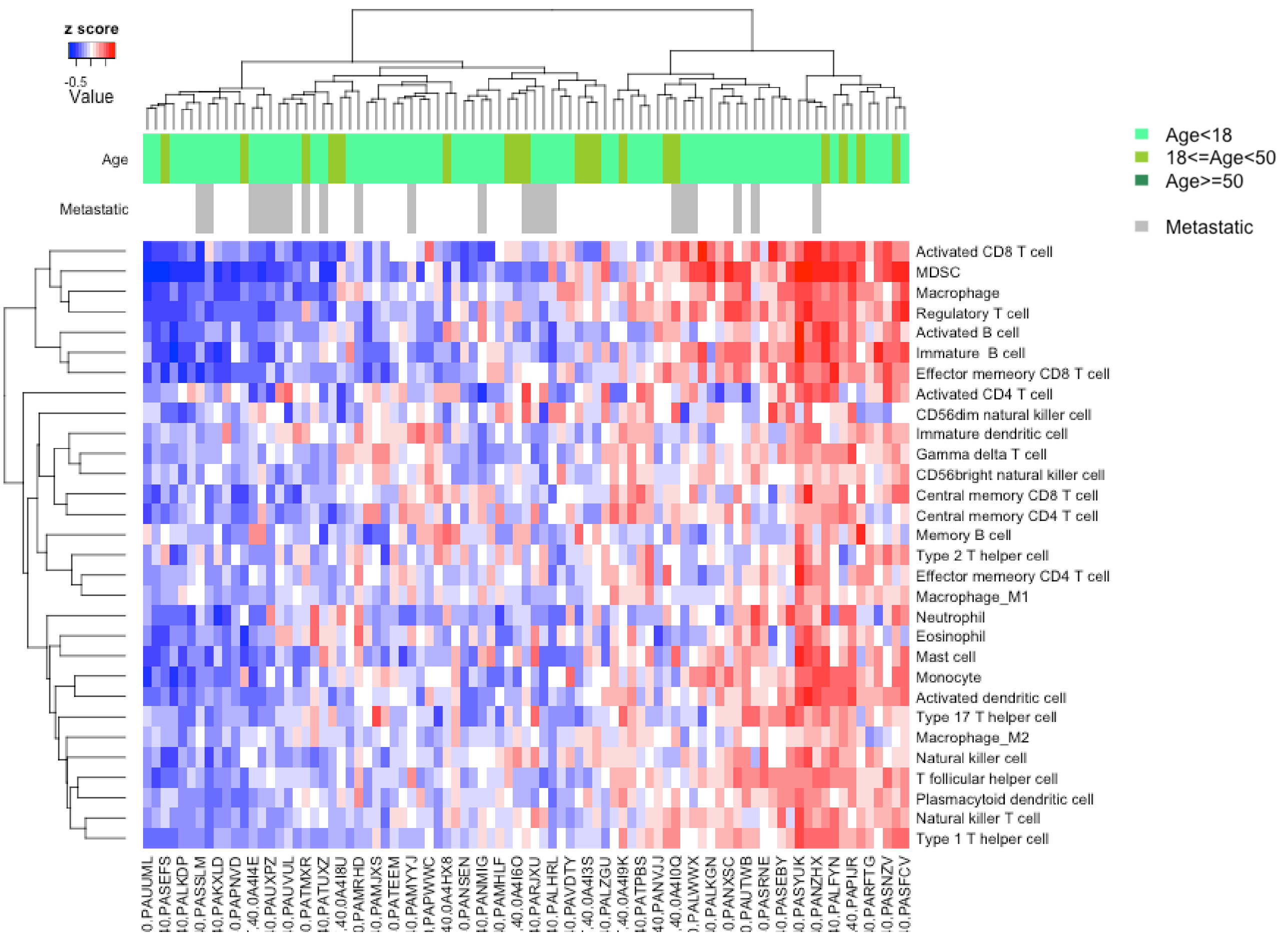

**Supplemental Figure 10.** TARGET immune score clustering. Hierarchical clustering heatmap of z-scored ssGSEA enrichment scores of immune cell gene signatures (level is depicted according to the color scale) across samples in the TARGET OS cohort with clinical annotations (patient age categories and metastatic).

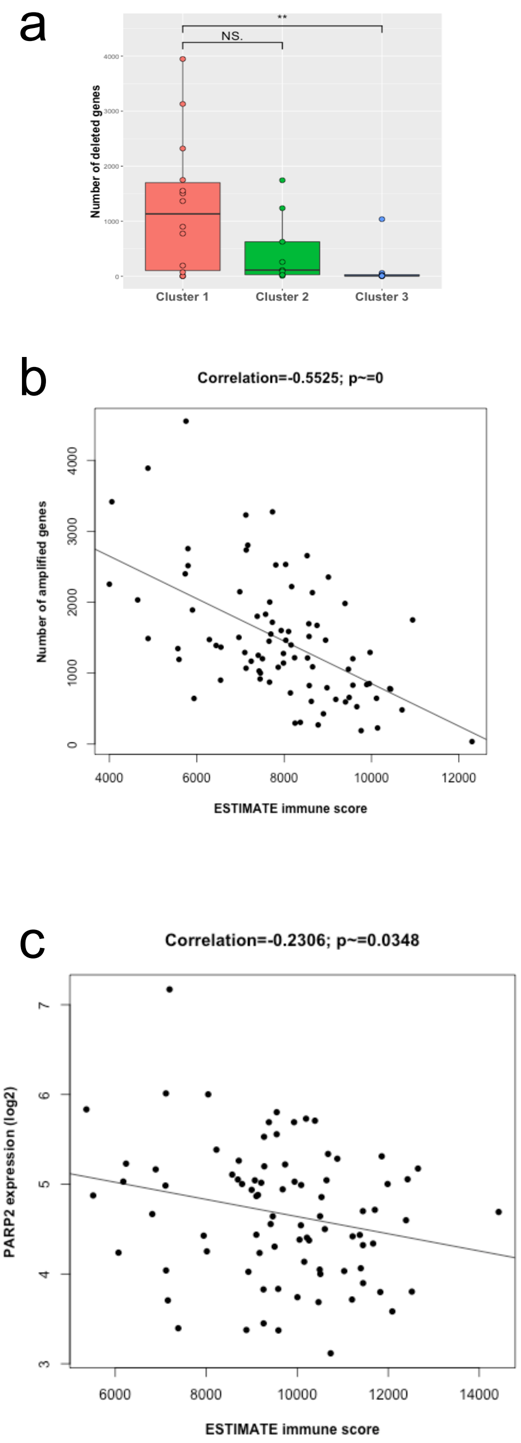
a
**
NS.
Number of deleted genes
4000
3000
2000
1000
0
Cluster 1
Cluster 2
Cluster 3
b
Correlation=-0.5525; p~=0
Number of amplified genes
0
1000
2000
3000
4000
4000
6000
8000
10000
12000
ESTIMATE immune score
c
Correlation=-0.2306; p~=0.0348
PARP2 expression (log2)
3
4
5
6
7
6000
8000
10000
12000
14000
ESTIMATE immune score

**Supplemental Figure 11.** Copy number alterations and immune infiltrate **a.** Boxplot of the number of deleted genes for each immune clusters C1, C2, and C3 (as described in Fig. 3b) for the MDACC cohort. The Wilcoxon rank sum test was used to determine significance of the comparison. **b.** Pearson correlation of the number of amplified genes and ESTIMATE immune score for the TARGET cohort. **c.** Pearson correlation of *PARP2* gene expression against ESTIMATE immune score for the TARGET cohort.

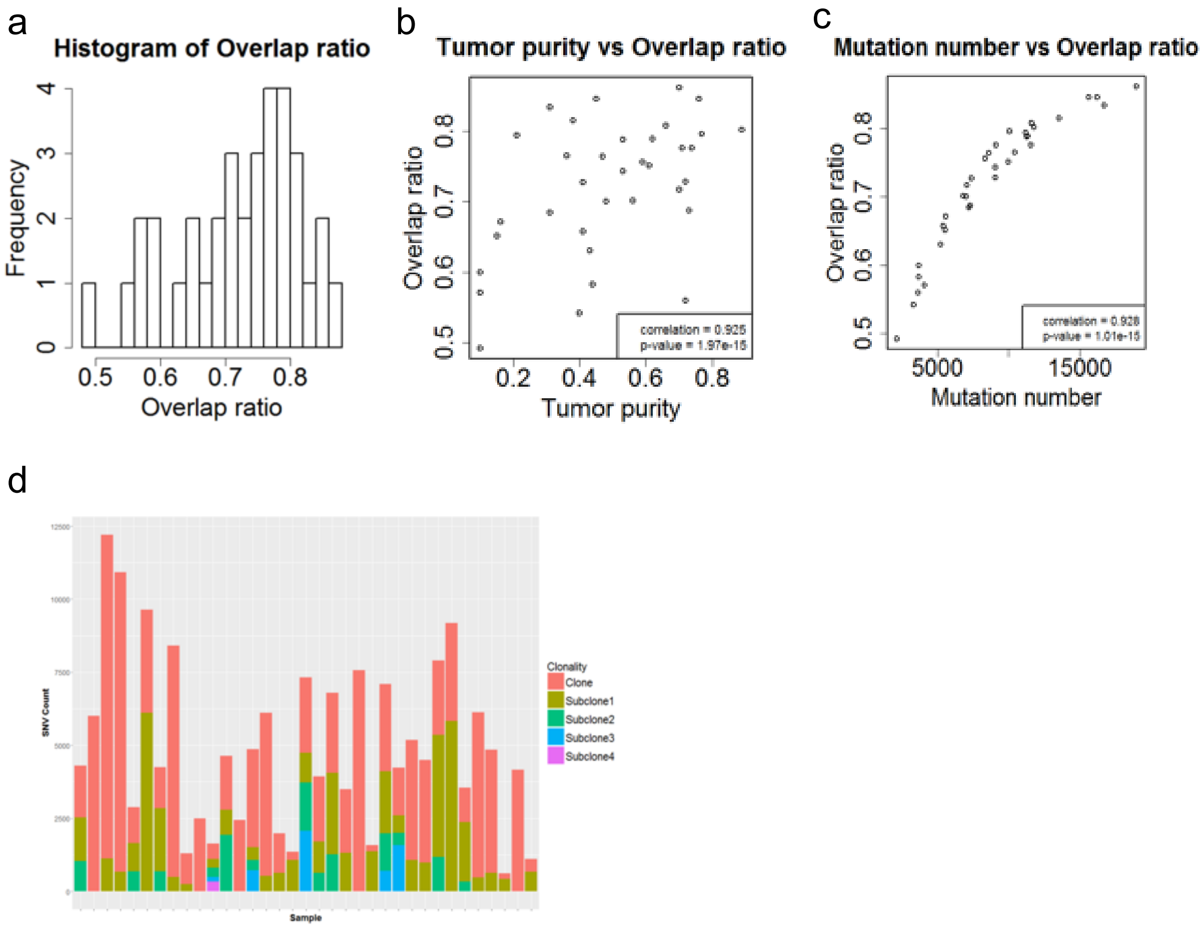
a
Histogram of Overlap ratio
Frequency
0
1
2
3
4
0.5
0.6
0.7
0.8
Overlap ratio
b
Tumor purity vs Overlap ratio
Overlap ratio
0.5
0.6
0.7
0.8
0.2
0.4
0.6
0.8
Tumor purity
correlation = 0.925
p-value = 1.97e-15
c
Mutation number vs Overlap ratio
Overlap ratio
0.5
0.6
0.7
0.8
5000
15000
Mutation number
correlation = 0.928
p-value = 1.01e-15
d
SNV Count
0
2500
5000
7500
10000
12500
Sample
Clonality
Clone
Subclone1
Subclone2
Subclone3
Subclone4

**Supplemental Figure 12.** Subclonal Architecture Analysis. **a.** Histogram of overlap ratio: the ratio of the number of consensus mutations (MuTect + MuSE) over the number of MuTect called mutations. **b.** Tumor purity vs overlap ratio. Tumor purity are estimated based on Sequenza. Each point represent a sample. The Pearson correlation and p-value of student’s t test indicate a significant association between tumor purity and overlap ratio. **c.** Mutation number vs overlap ratio. Mutation number are total somatic mutation number of consensus mutation between MuSE and MuTect. Each point represents a sample. The Pearson correlation and p-value of student’s t test indicate a significant association between consensus mutation number and overlap ratio. **d.** Clonality distribution of 35 samples. The length of the bar represents the consensus mutations.
