# Supplementary material for: Immuno-genomic landscape of osteosarcoma
Source: Nat Commun. 2020 Feb 21;11:1008. doi: 10.1038/s41467-020-14646-w (PMC7035358; doi:10.1038/s41467-020-14646-w)
Supplement: Supplementary file 26 — Reporting Summary [file 41467_2020_14646_MOESM26_ESM.pdf]

nature research

Corresponding author(s): P. Andrew Futreal

Last updated by author(s): Jan 10, 2020

Reporting Summary

Nature Research wishes to improve the reproducibility of the work that we publish. This form provides structure for consistency and transparency in reporting. For further information on Nature Research policies, see Authors & Referees and the Editorial Policy Checklist.

Statistics

For all statistical analyses, confirm that the following items are present in the figure legend, table legend, main text, or Methods section.

n/a	Confirmed	
☐	☒	The exact sample size (*n*) for each experimental group/condition, given as a discrete number and unit of measurement
☐	☒	A statement on whether measurements were taken from distinct samples or whether the same sample was measured repeatedly
☐	☒	The statistical test(s) used AND whether they are one- or two-sided<br>*Only common tests should be described solely by name; describe more complex techniques in the Methods section.*
☒	☐	A description of all covariates tested
☐	☒	A description of any assumptions or corrections, such as tests of normality and adjustment for multiple comparisons
☐	☒	A full description of the statistical parameters including central tendency (e.g. means) or other basic estimates (e.g. regression coefficient) AND variation (e.g. standard deviation) or associated estimates of uncertainty (e.g. confidence intervals)
☐	☒	For null hypothesis testing, the test statistic (e.g. *F*, *t*, *r*) with confidence intervals, effect sizes, degrees of freedom and *P* value noted<br>*Give P values as exact values whenever suitable.*
☒	☐	For Bayesian analysis, information on the choice of priors and Markov chain Monte Carlo settings
☒	☐	For hierarchical and complex designs, identification of the appropriate level for tests and full reporting of outcomes
☒	☐	Estimates of effect sizes (e.g. Cohen's *d*, Pearson's *r*), indicating how they were calculated

Our web collection on statistics for biologists contains articles on many of the points above.

Software and code

Policy information about availability of computer code

Data collection	No software was used.
Data analysis	CASAVA, BWA, BWA-MEM, Picard, GATK, STAR, HMMcopy, GISTIC 2.0, Sequenza, BRASS, MAPSPLICE, TelSeq, ClusteredMutations, CTLPScanner, ESTIMATE, TIMER, DAVID v.8, MuSE, MuTect, Pindel, CLiP, custom codes described in Supplemental Methods.

For manuscripts utilizing custom algorithms or software that are central to the research but not yet described in published literature, software must be made available to editors/reviewers. We strongly encourage code deposition in a community repository (e.g. GitHub). See the Nature Research guidelines for submitting code & software for further information.

Data

Policy information about availability of data

All manuscripts must include a data availability statement. This statement should provide the following information, where applicable:

- Accession codes, unique identifiers, or web links for publicly available datasets
- A list of figures that have associated raw data
- A description of any restrictions on data availability

The whole genome and transcriptome data generated during this study are available at the European Genome Archive under study EGAS00001003247. The TCR sequencing and RPPA data are available upon request. Upon request, Material Transfer Agreements will be completed before transfer of data.

Field-specific reporting

Please select the one below that is the best fit for your research. If you are not sure, read the appropriate sections before making your selection.

☒ Life sciences ☐ Behavioural & social sciences ☐ Ecological, evolutionary & environmental sciences

For a reference copy of the document with all sections, see nature.com/documents/nr-reporting-summary-flat.pdf

Life sciences study design

All studies must disclose on these points even when the disclosure is negative.

Sample size	We did not use any statistical methods to determine sample size. Since this is a rare tumor, we collected all possible samples and conducted analyses on all.
Data exclusions	No data were excluded.
Replication	Bioinformatic validations were conducted on independent datasets from the publicly available ICGC and TARGET cohorts as well as an internal clinical trial.
Randomization	This is not relevant to our study because it did not involve experimental groups.
Blinding	This is not relevant to our study because it did not involve experimental groups.

Reporting for specific materials, systems and methods

We require information from authors about some types of materials, experimental systems and methods used in many studies. Here, indicate whether each material, system or method listed is relevant to your study. If you are not sure if a list item applies to your research, read the appropriate section before selecting a response.

Materials & experimental systems

n/a	Involved in the study	
☐	☒	Antibodies
☒	☐	Eukaryotic cell lines
☒	☐	Palaeontology
☒	☐	Animals and other organisms
☐	☒	Human research participants
☐	☒	Clinical data

Methods

n/a	Involved in the study	
☒	☐	ChIP-seq
☒	☐	Flow cytometry
☒	☐	MRI-based neuroimaging

Antibodies

Antibodies used

RPPA CORE 05012017_133 (Set133); https://www.mdanderson.org/research/research-resources/core-facilities/functional-proteomics-rppa-core/antibody-information-and-protocols.html; CD3
Rabbit Polyclonal
1:100
Dako
CD4
4B12 (Mouse Monoclonal)
1:80
Leica (Novocastra)
CD8
C8/144B (Mouse Monoclonal)
1:25
Thermo Scientific
PD-1
EPR4877(2) (Rabbit Monoclonal)
1:250
Abcam
PD-L1
22C3 (Mouse Monoclonal)
Pre-dilute
Dako
Pax5
IEW (Mouse Monoclonal)
Pre-dilute

	Leica Biosystems<br>CD163<br>10D6 (Mouse Monoclonal)<br>1:100<br>Leica Biosystems<br>FoxP3<br>206D (Mouse Monoclonal)<br>1:50<br>Biolegend<br>CD73<br>D79A (Rabbit Monoclonal)<br>1:200<br>Cell Signaling
Validation	RPPA validation: Tibes, R. et al. Reverse phase protein array: validation of a novel proteomic technology and utility for analysis of primary leukemia specimens and hematopoietic stem cells. Mol. Cancer Ther. 5, 2512-2521 (2006); IHC validation by individual companies offering the antibodies.

Human research participants

Policy information about studies involving human research participants

Population characteristics	See Table 1 of the manuscript.
Recruitment	*Describe how participants were recruited. Outline any potential self-selection bias or other biases that may be present and how these are likely to impact results.*
Ethics oversight	University of Texas MD Anderson Cancer Center Institution Review Board

Note that full information on the approval of the study protocol must also be provided in the manuscript.

Clinical data

Policy information about clinical studies

All manuscripts should comply with the ICMJE guidelines for publication of clinical research and a completed CONSORT checklist must be included with all submissions.

Clinical trial registration	NCT02815995
Study protocol	https://clinicaltrials.gov/ct2/show/NCT02815995?term=NCT02815995&rank=1
Data collection	*Describe the settings and locales of data collection, noting the time periods of recruitment and data collection.*
Outcomes	*Describe how you pre-defined primary and secondary outcome measures and how you assessed these measures.*
